# Impact of school-based malaria case management on school attendance, health and education outcomes: a cluster randomised trial in southern Malawi

**DOI:** 10.1136/bmjgh-2019-001666

**Published:** 2020-01-14

**Authors:** Katherine E Halliday, Stefan S Witek-McManus, Charles Opondo, Austin Mtali, Elizabeth Allen, Andrew Bauleni, Saidi Ndau, Emmanuel Phondiwa, Doreen Ali, Virginia Kachigunda, John H Sande, Mpumulo Jawati, Allison Verney, Tiyese Chimuna, David Melody, Helen Moestue, Natalie Roschnik, Simon J Brooker, Don P Mathanga

**Affiliations:** 1 Department of Disease Control, London School of Hygiene and Tropical Medicine Faculty of Infectious and Tropical Diseases, London, UK; 2 Department of Medical Statistics, London School of Hygiene and Tropical Medicine Faculty of Epidemiology and Population Health, London, UK; 3 Save the Children Malawi, Zomba, Malawi; 4 Malaria Alert Centre, College of Medicine, University of Malawi, Blantyre, Malawi; 5 Zomba District Health Office, Ministry of Health, Zomba, Malawi; 6 District Education Office, Ministry of Education, Science and Technology, Zomba, Malawi; 7 National Malaria Control Programme, Ministry of Health, Lilongwe, Malawi; 8 Department of School Health, Nutrition, HIV & AIDS, Ministry of Education, Science and Technology, Lilongwe, Malawi; 9 Save the Children International, Blantyre, Malawi; 10 Save the Children International, Lilongwe, Malawi; 11 Save the Children, Washington, Washington DC, USA

**Keywords:** malaria, *Plasmodium falciparum*, rapid diagnostic tests, case management, teachers, schools, artemisinin-based combination therapy, Malawi, Africa

## Abstract

**Introduction:**

Evidence indicates children who suffer from ill-health are less likely to attend or complete schooling. Malaria is an important cause of morbidity and mortality in school-age children. However, they are less likely to receive malaria treatment at health facilities and evidence for how to improve schoolchildren’s access to care is limited. This study aimed to evaluate the impact of a programme of school-based malaria case management on schoolchildren’s attendance, health and education.

**Methods:**

A cluster randomised controlled trial was conducted in 58 primary schools in Zomba District, Malawi, 2011–2015. The intervention, implemented in 29 randomly selected schools, provided malaria rapid diagnostic tests and artemisinin-based combination therapy to diagnose and treat uncomplicated malaria as part of basic first aid kits known as ‘Learner Treatment Kits’ (LTK). The primary outcome was school attendance, assessed through teacher-recorded daily attendance registers and independent periodic attendance spot checks. Secondary outcomes included prevalence of *Plasmodium* spp infection, anaemia, educational performance, self-reported child well-being and health-seeking behaviour. A total of 9571 children from standards 1–7 were randomly selected for assessment of school attendance, with subsamples assessed for the secondary outcomes.

**Results:**

Between November 2013 and March 2015, 97 trained teachers in 29 schools provided 32 685 unique consultations. Female schoolchildren were significantly more likely than male to seek a consultation (unadjusted OR=1.78 (95% CI 1.58 to 2.00). No significant intervention effect was observed on the proportion of child-days recorded as absent in teacher registers (n=9017 OR=0.90 (95% CI 0.77 to 1.05), p=0.173) or of children absent during random school visits—spot checks (n=5791 OR=1.09 (95% CI 0.87 to 1.36), p=0.474). There was no significant impact on child-reported well-being, prevalence of *Plasmodium* spp, anaemia or education scores.

**Conclusion:**

Despite high community demand, the LTK programme did not reduce schoolchildren’s absenteeism or improve health or education outcomes in this study setting.

**Trial registration number:**

ClinicalTrials.gov NCT02213211.

**Key questions**

**What is already known?**

- Despite evidence that school-age children experience high burdens of *Plasmodium* infection, contributing to poor health and education outcomes, they are significantly less likely than any other age group to be prioritised for routine malaria control interventions.
- School-based administration of presumptive treatment for malaria has previously been demonstrated to be feasible and effective, but is no longer viable due to changes in global policy recommending parasitological confirmation of malaria prior to treatment in individuals over 5 years, and there is limited evidence on the effectiveness of such case management in schools.

**What are the new findings?**

- The introduction of school-based first aid kits, which included malaria case management with rapid diagnostic tests and artemisinin combination therapy, led to high levels of utilisation by schoolchildren.
- Despite high levels of uptake, the intervention did not lead to a significant reduction in school absenteeism or improvements in health and education outcomes in this setting in rural Malawi.

**What do the new findings imply?**

- School-based malaria case management is not an effective way to improve health and education outcomes in Malawi, but may provide a supplementary strategy to bolster health system efforts towards universal access to malaria diagnosis and treatment.
- Future research should focus on refining school-based approaches and how they can be effectively integrated alongside facility and community-based approaches to healthcare delivery.

## Introduction

Malaria is estimated to contribute between 5% to 8% of all-cause absenteeism among African schoolchildren, equivalent to 50% of all preventable absenteeism.^1^ Evidence from high transmission settings suggest each episode of clinical malaria is responsible for between 2.4 days and 6.5 days absenteeism from school^2–5^ and in western Kenya, malaria was reported by caregivers to account for over a third of school days missed.^6^ As well as a recognised contributor to anaemia among school-age children, from an educational perspective, malaria can have a direct impact on intellectual development in children through impaired attention and cognitive function.^7–11^ Despite this, school-age children are significantly less likely to sleep under a bednet, to seek treatment for malaria or to receive care from a formal source.^12–14^

A 2017 review described a range of strategies aimed at reducing the burden of malaria among school-aged children,^15 16^ including using antimalarial drugs for prevention, as well as case management within the school context.^7 17 18^ Case management evaluations in Ghana, Malawi and Tanzania demonstrated teachers can effectively deliver presumptive malaria treatment in schools.^19–21^ In Malawi, a school-based malaria case management intervention in which teachers were trained to presumptively treat malaria using sulfadoxine-pyrimethamine within a Pupil Treatment Kit (PTK) demonstrated declines in overall and malaria-specific mortality rates,^21^ as well as reductions in school grade repetition and absenteeism.^22^ However, national scale-up was curtailed in 2008 due to a change in first-line malaria treatment to artemisinin-based combination therapies (ACTs) and WHO recommendations that treatment should be preceded by parasitological confirmation, such as rapid diagnostic tests (RDTs).^23^

Over the past decade, extensive efforts have been invested globally into expanding the use of RDTs for malaria diagnosis beyond the formal health sector,^24^ incorporating both the private sector and community health workers (CHWs).^25^ A recent evaluation in Malawi found salaried CHWs using RDTs and ACTs to be feasible and effective providers of community-based case management of malaria for preschool-age children.^26^ This interest in expanding the use of RDTs across a broader range of providers developed alongside the commitment of the Malawian Ministry of Health (MoH) to providing universal access to prompt diagnosis and effective treatment^27^ and the prioritisation of increased enrolment and healthy children by the Ministry of Education Science and Technology (MoEST). This led to a multisectoral call for the re-introduction of school-based malaria case management, now incorporating RDTs and ACTs as part of a first aid kit operated by teachers.^28^ This study presents a cluster randomised evaluation of such an intervention on schoolchildren’s school attendance, health and education in southern Malawi.

## Methods

This trial is reported in accordance with the Consolidated Standards of Reporting Trials checklist (online supplementary Checklist S1) for cluster randomised trials, and the data, tools and associated material can be accessed through the London School of Hygiene & Tropical Medicine (LSHTM) Data Compass https://doi.org/10.17037/DATA.203. The protocol can also be found as online supplementary protocol S1.

10.1136/bmjgh-2019-001666.supp1 Supplementary data

10.1136/bmjgh-2019-001666.supp2 Supplementary data

### Study setting

The trial was conducted from 2011 to 2015 in Traditional Authority (TA) Chikowi in Zomba District, southern Malawi (online supplementary figure S1). The TA comprises a population of approximately 206 081.^29^ It is served by 58 public primary schools, all supported by Save the Children's Sponsorship Programming since 2008 with a package of school-based interventions addressing four key areas: early childhood care and development; basic education; school-health and nutrition; and adolescent development.^30^ Malaria remains the leading cause of morbidity and mortality in Malawi, with an estimated 2.1 million cases per year, within the age group of 5–14 years.^31^ The study area is characterised by intense transmission, with seasonal peaks during the rainy season (December to March).^32^ School-age children consistently exhibit the lowest bednet usage of all age groups, with usage reported at 42.0% in 2014 following a nationwide long-lasting insecticidal bednet distribution campaign in 2012.^12 33^

10.1136/bmjgh-2019-001666.supp3 Supplementary data

Despite the 2010 Malawi Demographic Health Survey indicating a relatively high net school attendance (enrolment) ratio,^29^ the primary completion rate is estimated at between 30% and 40%.^34^ Nationally, no difference in attendance, grade repetition or dropout is recorded between sexes until 15 years of age, after which the attendance rate is significantly higher in boys.^29^ The national pupil-teacher ratio is 74:1, compared with 76:1 in Zomba.^34^ A national survey of 1697 primary schools in 2011 reported that only 36% had a teacher trained in first aid, and that only 9% had a first aid kit available.^35^

### Study design

**Figure 1:**
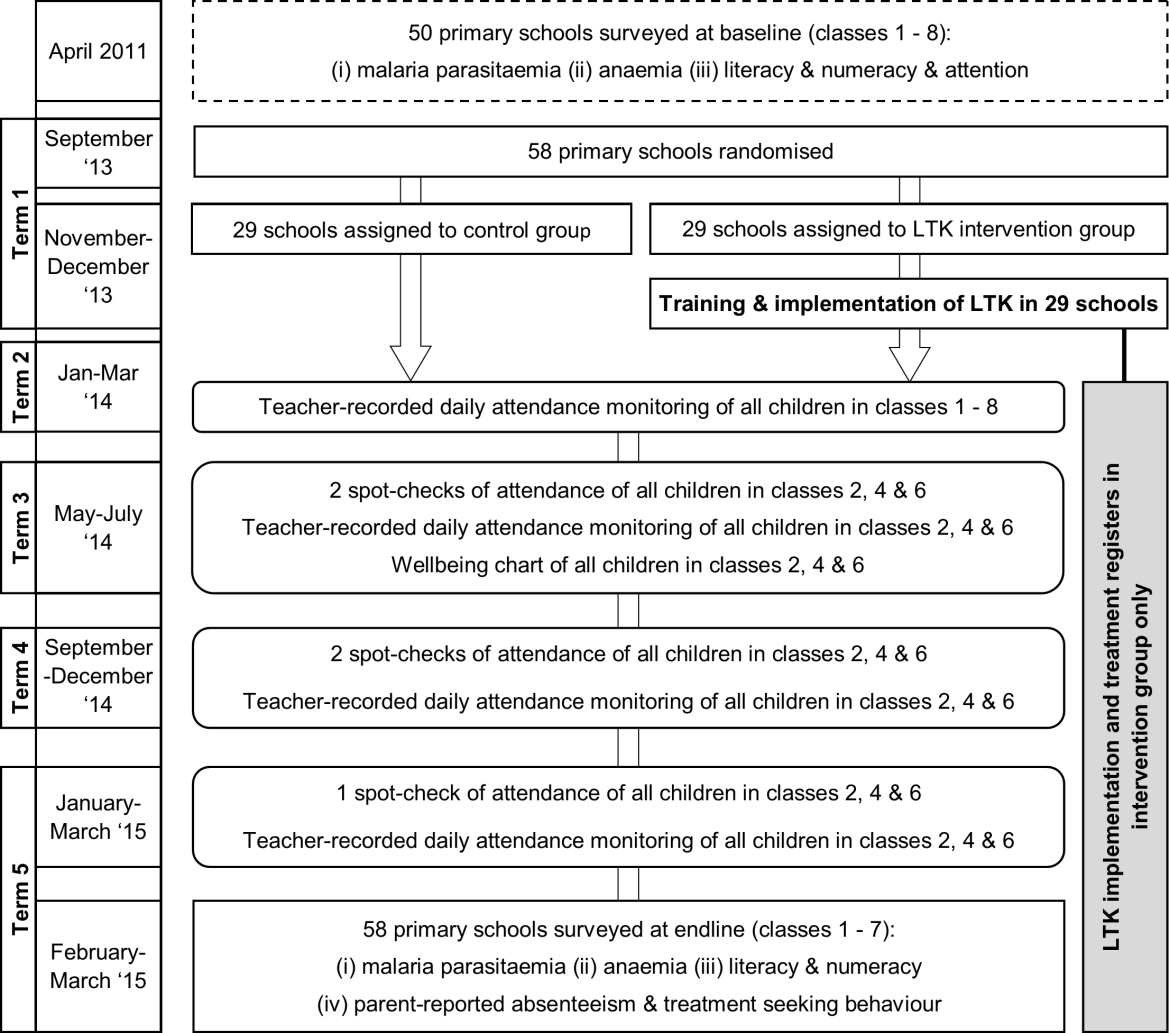
Study design and timeline. LTK, Learner Treatment Kit.

The impact of the intervention was evaluated through a cluster randomised controlled design (1:1) in which the 58 schools were randomised to one of two groups: (1) Learner Treatment Kit (LTK) intervention, whereby teachers were trained to diagnose uncomplicated malaria using RDTs and treat with ACTs, as well as to provide treatment for minor illness and injury, or to refer to the nearest health centre. (2) Control group, whereby no intervention was implemented. Due to the nature of the intervention, the study was not blinded. The primary outcome was school attendance, assessed through routine daily attendance registers and spot checks conducted across the 16-month implementation period. Secondary outcomes were measured through repeat cross-sectional surveys (at baseline in 2011 and at follow-up in 2015) and included malaria parasitaemia, anaemia, educational performance and parent-reported health-seeking behaviour assessed through surveys with a selection of children and their caregivers.^36^ Child-reported well-being was also recorded during the implementation period. Figure 1 shows the study design and timeline of the intervention and assessments.

### Sample size and randomisation

Power analysis was adjusted for clustering^37^ and focused on the percentage absenteeism as assessed by class attendance registers. Conservatively taking an intracluster correlation coefficient of 0.2, a baseline percentage absenteeism of 30% and 60 children (20 children from each of classes 2, 4 and 6) per school, a trial involving 29 schools per arm would provide 80% power to detect a difference of 16% in absenteeism between arms at the 5% significance level. If one school per arm were to be lost to follow-up over the course of the trial there would be 80% power to detect a difference of 17% in absenteeism.

Randomisation was stratified by school size (enrolment <1000 and ≥1000) and existing programmes or interventions which may affect school attendance, comprising three types of programmes; literacy and numeracy, early childhood development (nursery), or nutritional programmes. The random selection of children for assessment of attendance was performed by an external statistician, using computer-generated random number tables alongside the full enrolment list of children in classes 2, 4 and 6 in all 58 schools. Children were assessed against the following eligibility criteria for inclusion in the follow-up surveys: enrolled in classes 2, 4 and 6 of participating schools at the start of the intervention; provision of informed consent from a caregiver; and provision of assent by the child. Due to the high rate of dropout expected in schools within the year, 16 reserve children were selected per class (where available) in addition to the 20 initially sampled, to account for loss to follow-up across the 16-month implementation period. All selected children were included in the measures of attendance. Separately, a further 16 children from each of classes 1, 3, 5 and 7 at the start of the intervention were also randomly selected for inclusion in the term 1 teacher-recorded daily attendance registers. Health and education surveys were conducted with the original 20 children in classes 2, 4 and 6 and four children from each of classes 1, 3, 5 and 7.

### Intervention

Full details of the intervention are described elsewhere,^28^ but in brief the LTK is a school-based first aid kit for the management of basic health problems, including uncomplicated malaria, basic illnesses (eg, headache, diarrhoea or vomiting) and minor injuries (eg, wounds or burns), available to all enrolled children in school (5–18 years), free of charge during school hours. At each intervention school, two to four teachers (dependent on school enrolment) received 7 days of training in the use of the LTK as an ‘LTK Dispenser’, alongside a manual, job aid and three half days of mentoring at a local health centre. Ongoing supervision from the district health office and district education office was conducted, with additional support from the two ministries at the National level and Save the Children. A 2-day refresher training was held midway through the 16-month implementation period. LTK supplies were locked in a wooden box, for which only LTK dispensers and head teachers had keys. In addition to RDTs, ACTs and materials related to malaria testing and treatment, the LTK included basic supplies such as oral rehydration solution and bandages. Non-medical biowaste was burned on school grounds. Sharps and biowaste were disposed of in specially constructed locked pit latrines, separate to latrines used by children. LTK supplies were stored at assigned nearby primary healthcare centres where they could be requested and collected by the head teacher.

Children were informed that whenever they felt unwell, they could report to an LTK dispenser during break time for assistance. LTK dispensers followed a simple algorithm, recording symptoms reported and the actions taken in a treatment register. In the event of any complicated or urgent health complaint, the child’s caregiver was contacted and the child immediately referred to a local health centre.

Following the baseline surveys in 2011, the LTK intervention and training materials were refined across a series of stakeholder meetings between June 2012 and June 2013 at which point they were tested at a pilot training.^28^ Subsequently 97 teachers were trained for the trial in November and December 2013 (29 head teachers and 68 teachers). This development and piloting of the intervention had the consequence of extending the time between baseline and follow-up surveys. The LTK dispensers received regular supervision and monitoring visits from the study team, during which supplies were checked, referral forms reviewed, and protocols and treatment registers verified through direct observation of schoolchildren receiving a consultation. Overall, implementation ran between December 2013 and March 2015 in the schools, covering two rainy seasons.

A passive surveillance system based on routine reporting systems used by the MoEST was used to monitor adverse events. A simultaneous active surveillance system whereby study officers interviewed head teachers on a termly basis was also established. Where possible, reported cause of death was recorded using head teachers’ reports, as death certificates were frequently unavailable.

### Assessments

The primary outcome (school attendance) was measured in all schools using two methods: daily attendance rate and attendance during unannounced spot check visits, both assessed via days absent. Daily attendance was recorded by the class teachers on standardised 5-week long class registers generated using full enrolment lists for each school. These were distributed to the teachers to be filled in daily for each child and were collected in by the study team. Spot check attendance was assessed through unannounced school visits by study officers at least once a term for children being followed from classes 2, 4 and 6 (a maximum of five rounds) in which presence or absence was documented for every child in the class. As the intervention took place across two academic years they were followed into classes 3, 5 and 7, respectively, as they moved up over the course of the study. If the child was absent, a specific absentee code was assigned based on the reason for absence reported by siblings, classmates or teacher. Transfers and dropouts were tracked through the daily attendance registers and the spot check visits.

Intervention uptake was assessed through retrospective review of the LTK treatment registers including date of consultation, demographic information, symptoms, test and treatment decisions. Malaria parasitaemia and anaemia were measured at baseline and follow-up surveys. A finger-prick blood sample was used to assess haemoglobin concentration using a portable photometer (Hemocue, Ängelholm, Sweden), and to prepare thin and thick films for confirmation and quantification of malaria parasites on the basis of expert microscopy.

Education outcomes were assessed prior to the intervention using tests of sustained attention, literacy and numeracy. Further details of these test procedures at baseline are provided elsewhere.^36^ During the follow-up surveys literacy and numeracy were assessed through tests administered at the group level. Numeracy was assessed through a missing number and arithmetic task in the lower classes (1 to 4),^38^ and a written arithmetic assessment in the upper classes (5 to 8). With regard to literacy, spelling tasks were adapted from the Phonological Awareness Literacy Screening tool.^39^

Child-reported well-being was assessed in all schools. Charts were placed on the walls listing every child in the class, with dates. On Mondays, Wednesdays and Fridays children were required to draw a happy, neutral or sad face next to their name depending on whether they felt well, average or ill. If the child was absent that day, a cross was put in the box for that child by the teacher or a classmate. The charts were piloted prior to use to ensure standardised meaning was applied to the three types of faces.

Caregivers of children included in the follow-up surveys were interviewed on sociodemographic information, in addition to treatment-seeking behaviour for their children and days of school lost during their children’s last bout of illness (for those who reported their child had been absent due to illness in the last 2 weeks).

### Data analysis

Primary analyses were conducted using the intention-to-treat principle. Primary and secondary outcomes were prespecified and approved by an independent data monitoring committee. Data from the 36 children randomly selected from classes 2, 4 and 6 in the 58 schools were used to evaluate effectiveness of the LTK intervention regarding school attendance via independent spot checks and teacher-recorded daily attendance measures. The additional 16 children from each of classes 1, 3, 5 and 7 were assessed for school attendance using the teacher-recorded daily attendance measure only. The health and education outcomes at follow-up were assessed with the original 20 children selected from classes 2, 4 and 6, as specified in the sample size calculation and with an additional four children from each of classes 1, 3, 5 and 7. Children no longer available or eligible were replaced with children from the reserve list.

Teacher-recorded daily attendance was defined as the proportion of missed school days (absent) of those eligible for measurement in the daily attendance registers. Once the child was recorded as having left or transferred out of the school, their follow-up time was censored. The spot check attendance was defined as a binary outcome (present/absent) at each of the five spot check visits. Well-being was coded as an ordinal variable: well, average, not well. Anaemia was defined according to WHO age-sex-specific cut-offs for haemoglobin (g/L).^40^ Malarial parasitaemia was characterised as presence or absence of parasites as well as parasite density for positive individuals. Age in years, and categorised into groups, was derived using child-reported information during enrolment. Scores for the two education outcomes (literacy and numeracy) were standardised separately by class, permitting combined statistical analysis across classes, thereby simultaneously controlling for both class and test version. Non-normality of the cognitive and educational scores was addressed through bootstrapping.^36 41^

The study population assessed at baseline (2011) was different to the population followed during the implementation period (2014–2015) and assessed at follow-up, thus analysis of baseline characteristics was made only for study-arm comparisons. All analyses estimated unadjusted and adjusted measures of effect and 95% CIs. Adjustment was made for child’s age and sex, and school-level stratification factors. Additional adjustment was made for school-level flooding in January and February 2015 (which may have impacted on attendance due to school closures). Multilevel logistic regression models were used to estimate the ORs for absenteeism between children in the two arms of the trial for both attendance measures. All models allowed for clustering within schools and, where appropriate, clustering within individuals for repeated measures data. The proportions of missed school days in the daily attendance registers were estimated at the individual level. Multilevel linear regression models with random effects at the school level were used to assess differences in educational outcomes, parasite prevalence and density, and haemoglobin level. For child-reported well-being, a multilevel ordinal logistic regression of the repeated within-child measures with a random effect at the school level was used.

### Patient and public involvement

Although community members were not directly involved in establishing the research question or evaluation outcomes, prior to starting activities, school-level meetings were held with community members and school authorities to explain the proposed intervention and evaluation procedures. Any questions, concerns or suggestions arising were discussed and incorporated, when appropriate, into the intervention and study procedures. Additionally, similar school meetings were held routinely throughout the study period.

### Ethics statement

As the intervention comprised a school-based health service, consent was given at the school level by the head teacher, parent teacher association and school management committee at the school-level meetings. All children in intervention schools were eligible to seek treatment and caregivers could opt out of the intervention on their child’s behalf. Informed consent was obtained prior to training teachers as LTK dispensers. Written informed consent was obtained from the caregivers of children randomly selected to participate in the baseline and follow-up assessments, as well as written assent from the children.

## Results

### Baseline characteristics and trial profile

Children assessed from the 50 schools in 2011 were broadly similar between study groups with regards to age, sex, anthropometric indices, bednet use and household characteristics. Slight differences were observed in some school characteristics (school size and school feeding programmes) and household-level socioeconomic status (SES) (table 1). While anaemia and educational outcome measures were similar between groups, there was an imbalance in the prevalence of *Plasmodium falciparum* infection 56.4% and 63.0% in the control and intervention groups, respectively. No measure of school attendance was available at baseline.

**Table 1:** Baseline school, child and household characteristics by study group for 2667 children

Characteristic; n (%) *	Control	Intervention
School characteristics †	23 schools	27 schools
Children sampled per school		
No. of children sampled, median (range)	55 (31–64)	56 (22–65)
School size (enrolment)		
No. of children enrolled, mean (SD)	1140.30 (550.03)	968.71 (375.69)
School programme		
Feeding	2 (8.7%)	6 (22.2%)
Deworming	16 (72.7%)	20 (74.1%)
Malaria control	12 (54.5%)	9 (33.3%)
School facilities		
Water and sanitation	7 (31.8%)	7 (25.9%)
Gender-separated toilets	20 (90.9%)	23 (85.2%)
Hand-washing facilities	8 (36.4%)	14 (51.9%)
Child characteristics †	1224 children	1443 children
Age in years		
Mean (SD)	11.72 (3.17)	11.88 (3.13)
5–9	313 (25.6%)	347 (24.1%)
10–12	372 (30.4%)	430 (29.8%)
13 or more	539 (44.0%)	666 (46.2%)
Sex		
Male	587 (48.0%)	684 (47.4%)
Female	637 (52.0%)	759 (52.6%)
Anthropometric z scores		
Weight for age, mean (SD)	−1.03 (1.06)	−1.12 (1.18)
Height for age, mean (SD)	−1.31 (1.25)	−1.47 (1.22)
Body mass index for age, mean (SD)	−0.67 (1.02)	−0.67 (0.85)
Nutritional status		
Underweight	66 (16.8%)	80 (18.4%)
Stunted	308 (25.4%)	460 (32.1%)
Thin	76 (6.3%)	93 (6.5%)
Health status		
No. with *Plasmodium falciparum* infection (%)	692 (56.4%)	909 (63.0%)
Parasite density parasites/µl, mean (SD)	716.76 (2832.41)	743.80 (2225.41)
No. of anaemic children (%)	388 (31.7%)	476 (33.0%)
Haemoglobin concentration g/L, mean (SD)	124.87 (15.12)	125.24 (15.98)
Bednet use		
Used a bednet last night (%)	414 (33.9%)	446 (31.0%)
Educational assessments		
Sustained attention score, mean (SD)	0.03 (1.01)	−0.03 (0.99)
Numeracy test score, mean (SD)	0.07 (1.02)	−0.06 (0.98)
Literacy test score, mean (SD)	−0.03 (1.05)	0.03 (0.96)
Household characteristics †		
Parental education		
No schooling	195 (16.9%)	241 (17.7%)
Primary schooling	788 (68.2%)	970 (71.1%)
Secondary schooling or higher	173 (15.0%)	153 (11.2%)
Socioeconomic status		
Poorest	221 (20.0%)	282 (21.0%)
Poor	243 (22.0%)	261 (19.4%)
Median	199 (18.0%)	323 (24.0%)
Less poor	201 (18.2%)	249 (18.5%)
Least poor	243 (22.0%)	232 (17.2%)

*% of non-missing children in each study group presented for categorised data. For continuous data mean (SD) is presented

†All characteristics have less than 2% missing data with the exception of following indicators: stunted, thin and underweight.

SD, standard deviation.

**Figure 2:**
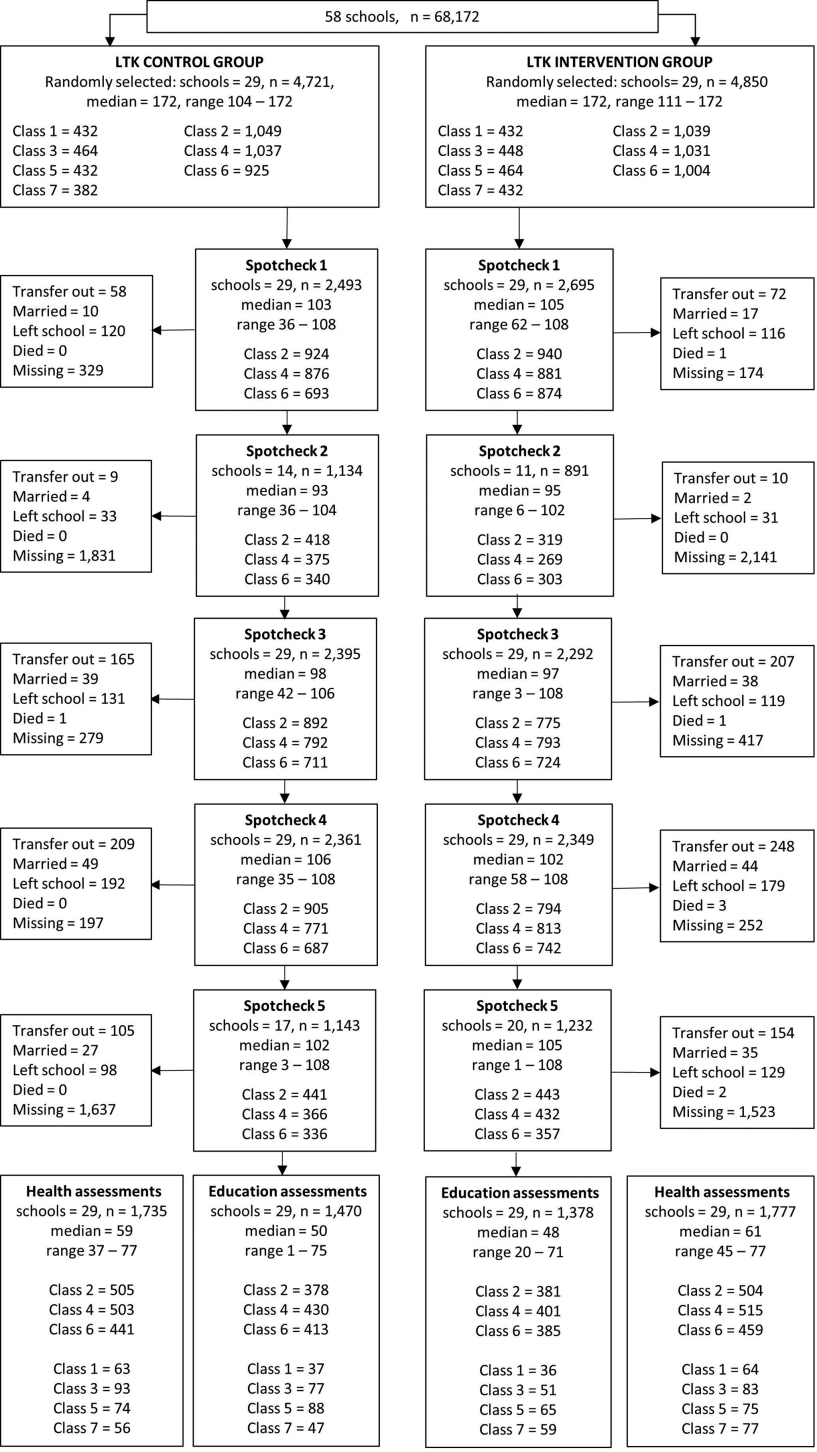
Trial participant flow diagram. LTK, Learner Treatment Kit: Fifty-eight schools were randomised to either receive the LTK programme or act as controls. No schools discontinued the intervention. Only classes 2, 4 and 6 were included in the spot check assessments and were included in the follow-up education and parasitology assessments. The additional children from classes 1, 3, 5 and 7 were included in the teacher-recorded registers in term 1 and a subsample was included in the follow-up education and parasitology assessments

In total 9571 children were randomly selected from classes 1 to 7 of the school enrolment lists in December 2013 (figure 2). In classes 2, 4 and 6, the school attendance of 3011 and 3074 selected children from the control and intervention groups, respectively, were followed up at a maximum of five spot checks. Due to turnover of students, attendance was recorded for 82.8% and 87.9% of the selected children in the control and intervention groups, respectively, at spot check 1. All schools were visited during spot checks 1, 3 and 4; and half of the schools were visited at spot checks 2 and 5. Daily attendance data were available for 9023 (94.3%) of the sampled children for at least one 5-week block of attendance. The number of children included per school for this outcome ranged between 97 and 171.

In March 2015, the follow-up health and education assessments were conducted with 3512 children. The numbers of children assessed at follow-up per school ranged from 20 to 77 (with the exception of one school in which only 1 child was assessed for education outcomes) but overall numbers were well balanced between groups (figure 2).

### Intervention compliance and uptake

Overall, retention of trained teachers to the programme was high. Of the original 97 teachers trained, 3 were transferred to schools outside of the study area and 1 declined to continue as an LTK dispenser. A replacement training for these four teachers took place during August 2014. Following this a further seven teachers transferred out of their school, but were not replaced.

A summary of LTK usage and uptake is detailed in table 2. The number of LTK consultations varied significantly by transmission season, with over twice as many consultations sought during the first rainy season than the post-rainy and dry season, followed by another increase in consultations observed in the second rainy season. Of those consulting an LTK dispenser, on average 72% were eligible for an RDT (based on symptoms reported, that is, reported fever in past 72 hours, plus one or more symptoms such as: headache, stomach ache, nausea, diarrhoea, vomiting) during the first rainy season and the post-rainy season, which declined to below 60% during both the dry and second rainy seasons. The highest RDT positivity rate was seen in the post-rainy season (64.9% of those consulted and 81.7% of those tested) and the lowest was observed during the dry season (35.8% of those consulted and 58.9% of those tested). During both rainy seasons, over 70% of those tested with an RDT received a positive result.

**Table 2:** Summary of LTK consultations by school term

**School term**	**Season**	**N total consultations**	N (%) eligible for RDT (i.e., reporting fever +1 relevant symptom)*	**N (%) tested with RDT†**	**N (%) confirmed malaria positive by RDT‡** **[test positivity rate**]	**N (%) treated with AL§ [% of confirmed cases treated**]
Dec-Mar 2014¶	Rainy	12 654	9074 (71.7)	9909 (78.3)	7651 (60.5) [77.2]	7388 (58.5) [96.6]
May-Jul 2014	Post-rainy	5665	4154 (73.3)	4503 (79.5)	3678 (64.9) [81.7]	3535 (62.4) [96.1]
Sept-Nov 2014	Dry	5772	2996 (51.9)	3510 (60.8)	2068 (35.8) [58.9]	1956 (33.9) [94.6]
Dec-Mar 2015	Rainy	8594	5082 (59.1)	5608 (65.3)	4157 (48.4) [74.1]	3876 (45.1) [93.2]
**Totals**		32 685	**21 306** (**65.2**)	**23 530** (**72.0**)	**17 554 (53.7) [74.6**]	**16 755** (**51.3**) **[95.4]**

Total number of unique consultations, number eligible (ie, reporting relevant symptoms) for an RDT, number tested by RDT, number testing malaria-positive by RDT, number treated with ACT for RDT and tested by RDT; displayed as a percentage of all unique consultations. Numbers in square brackets denote test positivity rate and percentage of confirmed cases treated.

*Includes those eligible for RDT who reported fever plus any of headache, vomiting, diarrhoea, dehydration, nausea, stomach ache, weakness and cough; excludes those eligible for RDT who reported fever plus only general aches, muscle/joint pains or loss of appetite.

†Includes those tested in the absence of fever plus one other relevant symptom (n=2969, 12.6%) and those presumed to be tested by RDT—where RDT results are recorded but data for RDT performed are missing (n=187, 0.6%).

‡Includes those presumed to be positive by RDT—where RDT was performed and treatment was recorded but data for RDT results are missing.

§Includes those treated in the absence of RDT conducted, and those treated with RDT-negative result.

¶Includes consultations conducted in November 2013 (n=499).

AL, artemether lumefantrine; LTK, Learner Treatment Kit; RDT, rapid diagnostic test.

Across all children attending the intervention schools, there were substantially more consultations provided to female schoolchildren aged 6–14 years than to boys for any reason (64.9% *vs* 35.1%), despite girls comprising half (49.4%–51.1%) of all enrolled children in this age group. Repeat consultations within the 16-month implementation period were recorded. Within the 4850 participants randomly selected from the intervention schools (figure 1) girls had nearly twice the odds of attending at least one consultation as boys (unadjusted OR =1.78 (95% CI 1.58 to 2.00), p≤0.001) (table 3).

**Table 3:** Consultations attended by boys and girls

**Consultations attended**	**Boys** n*=2430	**Girls** n*=2420	**Unadjusted**		**Adjusted**	
			**OR† (95% CI**)	**P value**	**OR† (95% CI**)	**P value**
Children attending at least one consultation, total	846	1165	1.78 (1.58 to 2.00)	<0.001	1.81 (1.60 to 2.05)	<0.001
Consultations per child, mean (SE)	0.63 (1.17)	1.02 (1.54)
Overall number of consultations attended	1527	2471				

Numbers, unadjusted and adjusted ORs for attending at least one consultation by girls relative to boys in the randomly sampled children followed throughout implementation.

Unadjusted: All children with outcome measures, not adjusted for any demographic or study design characteristics.

Adjusted: for age, school size, school flooding and school programmes considered in stratification and for school flooding in January 2015.

*n = number of children offered consultations (not withdrawn or deceased)

†Standard errors adjusted for clustering within schools

SE, standard error.

The most commonly reported symptom across all children seeking an LTK consultation was headache, recorded in a third of all consultations. One in four children reported fever in the past 72 hours, and one in five reported abdominal pain. Wounds accounted for 1% of all consultations. Despite the greater number of girls seeking treatment than boys, the range of symptoms reported were similar between sexes.

### Effect of the LTK intervention on attendance

There was no significant difference observed for the primary outcome of attendance (days absent) between study groups as measured by either teacher-recorded daily registers in the wider sample (OR 0.90 (95% CI 0.77 to 1.05)) or field officer-recorded spot checks in the sample of children from classes 2, 4 and 6 (OR 1.09 (95% CI 0.87 to 1.36)). The adjusted analyses were consistent, as shown in table 4.

**Table 4:** Effect of the LTK intervention on incidence of absenteeism of schoolchildren throughout the study period

Primary outcomes	Control (n*=29)		Intervention (n*=29)		Unadjusted		Adjusted	
	n†	% Absent	n†	% Absent	OR‡ (95% CI)	P value	OR‡ (95% CI)	P value
Daily attendance	4430	20.86	4587	19.31	0.90 (0.77 to 1.05)	0.173	0.91 (0.78 to 1.06)	0.224
Spot checks	2867	22.22	2924	24.00	1.09 (0.87 to 1.36)	0.474	1.10 (0.88 to 1.39)	0.390

As measured by daily registers and periodic spot checks. Results presented (1) For all children with outcome data (unadjusted). (2) Once adjusted for age, sex and stratification effects as the primary prespecified analyses.

Unadjusted All children with outcome measures, not adjusted for any demographic or study design characteristics.

Adjusted for age, sex, school size and school programmes considered in stratification and for school flooding in January 2015

*Number of schools

†Number of children eligible for follow up (not withdrawn or deceased)

‡Standard errors adjusted for clustering within schools

LTK, Learner Treatment Kits.

Absenteeism varied between 16% and 32% across the five time points (online supplementary table S1). In both arms, absenteeism was consistently highest during the post-rainy (May–June 2014) and rainy seasons (January–February 2015), and lowest in the dry season. Absenteeism did not significantly differ at any of the five time point between arms. There was evidence of an interaction between the intervention and spot check time points (p=0.012) suggesting a variation in the impact of the intervention over time, but no strong systematic pattern was observed in this variation. Despite the greater intervention uptake in girls than boys, no differential impact between girls and boys was observed for either the daily monitoring (p=0.245) or the spot checks (p=0.481). Similarly, no heterogeneity in impact was observed across classes. However, a sensitivity analysis of absenteeism attributed to illness (as reported by the class teacher or another child in the class during the spot checks) revealed an increase in the intervention group (OR 1.68, 95% CI 1.24 to 2.29, p=0.001), although these reports were not subsequently validated by the child when they returned to school.

### Effect of the LTK intervention on child-reported well-being

There was no significant impact of the LTK intervention on child-reported well-being, measured from May to July 2014 (table 5). Children reported themselves as ‘feeling well’ 72% and 75% of the days; ‘feeling average’ 20% and 17% of the days and ‘feeling not well’ on 8% and 8% of days in the control and intervention groups, respectively (proportional OR 0.86, 95% CI 0.56 to 1.30).

**Table 5:** Effect of the Learner Treatment Kit (LTK) intervention on child-reported well-being during June–July 2014 for study children

**Secondary outcomes**	Control (n*=29)		Intervention (n*=29)		**Unadjusted**		**Adjusted**	
	**n days**	**%**	**n days**	**%**	**OR† (95% CI**)	**P value**	**OR† (95% CI**)	**P value**
Child-reported well-being‡								
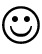 well	11 368	72.16	11 731	75.33	0.86 (0.56 to 1.30)§	0.471	0.85 (0.57 to 1.25)§	0.398
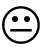 average	3109	19.73	2634	16.91				
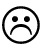 not well	1277	8.11	1207	7.75				

Unadjusted All children with outcome measures, not adjusted for any demographic or study design characteristics.

Adjusted for age, sex, school size and school programmes considered in stratification and for school flooding in January 2015.

*Number of schools

†Standard errors adjusted for clustering within schools.

‡There are up to 33 repeated measurements per child; one control cluster has no observations.

§Proportional OR.

### Effect of the LTK intervention on anaemia, parasitaemia, educational achievement, parent-reported absenteeism and treatment-seeking behaviour

At 16 months follow-up, 1735 children in the control schools and 1777 children in the intervention schools provided a finger-prick blood sample for the measurement of haemoglobin concentration and *Plasmodium* parasites (figure 2). There was no significant difference in either the prevalence of anaemia or *Plasmodium* infection between children in the two groups (table 6). Similarly, no effect of the LTK intervention was observed on education outcomes of literacy (p=0.471) and numeracy (p=0.921) at follow-up (table 6).

**Table 6:** Effect of the LTK intervention at 16 months follow-up on health and educational outcomes for study children as well as parent-reported absenteeism due to illness

Secondary outcomes	Control (n=29)*		Intervention (n=29)*		Unadjusted		Adjusted	
	n†	%	n†	%	OR‡ (95% CI)	P value	OR‡ (95% CI)	P value
Anaemia	1735	16.48	1776	17.57	1.07 (0.82 to 1.39)	0.613	1.06 (0.81 to 1.38)	0.670
Infection with *Plasmodium*	1732	16.40	1772	15.52	1.01 (0.65 to 1.57)	0.948	1.02 (0.71 to 1.47)	0.899

	n†	Mean (SD)	n†	Mean (SD)	Mean difference‡ (95% CI)	P value	Mean difference‡ (95% CI)	P value
Haemoglobin (g/L)	1736	129.33 (12.52)	1776	129.19 (12.64)	−0.15 (−1.73 to 1.44)	0.855	−0.38 (−1.95 to 1.18)	0.630
Parasite density (log count)	276	6.98 (1.41)	266	6.82 (1.47)	−0.18 (−0.46 to 0.09)	0.197	−0.18 (−0.44 to 0.08)	0.175
Standardised literacy score	1394	0.02 (1.01)	1352	−0.02 (0.99)	−0.07 (−0.27 to 0.12)	0.471	−0.09 (−0.27 to 0.10)	0.351
Standardised numeracy score	1438	0.00 (1.02)	1347	0.00 (0.98)	−0.01 (−0.22 to 0.20)	0.921	−0.03 (−0.22 to 0.16)	0.753
Parent-reported attendance (days absent)§	236	2.68 (2.02)	233	2.46 (1.71)	−0.09 (−0.57 to 0.40)	0.729	−0.08 (−0.55 to 0.40)	0.744

Unadjusted All children with outcome measures, not adjusted for any demographic or study design characteristics.

Adjusted for age, sex, school size and school programmes considered in stratification and for school flooding in January 2015.

*Number of schools.

†Number of children eligible for follow up (not withdrawn or deceased).

‡standard errors adjusted for clustering within schools.

§Days of school lost during their children’s last bout of illness (for those who reported their child had been absent due to illness in the last 2 weeks).

LTK, Learner Treatment Kit.

**Figure 3:**
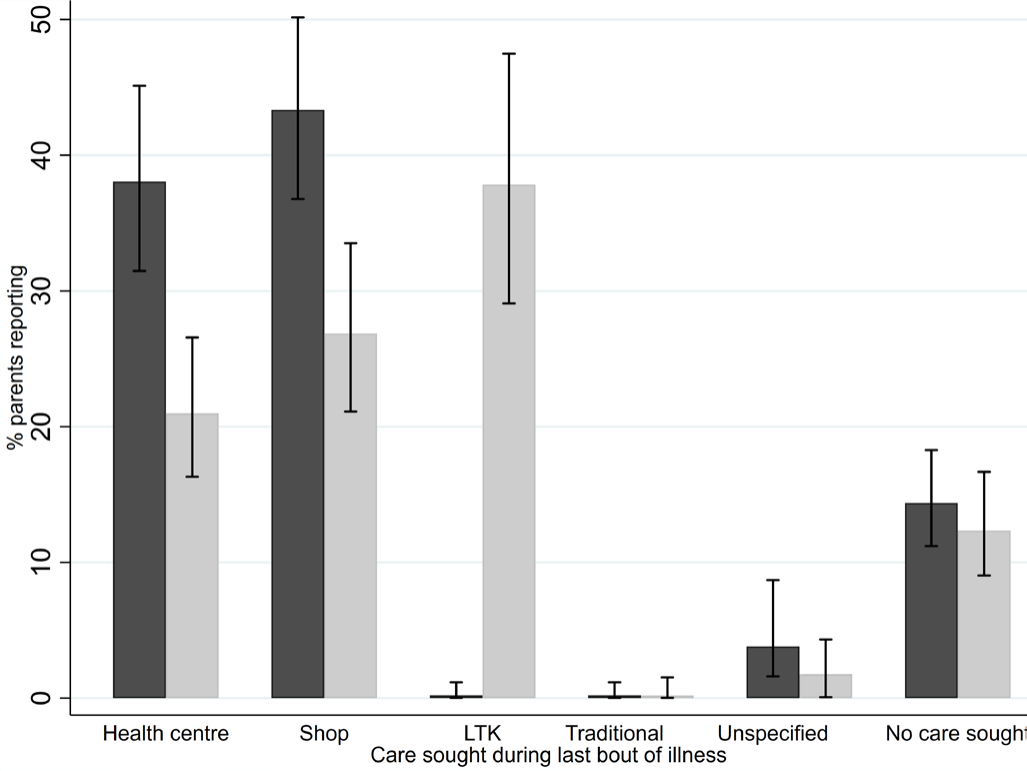
Effect on parent-reported health-seeking behaviour: 983 parents (473 in the control group (dark bars) and 510 in the intervention group (light bars)) interviewed at the follow-up survey on treatment-seeking behaviour for their children. 95% CI is denoted. LTK, Learner Treatment Kit.

No difference in parent-reported absenteeism was observed. A total of 983 parents were interviewed on treatment-seeking and a significant difference (p<0.001) was observed in the health-seeking behaviour of parents with children between study groups with fewer parents in the intervention schools reporting seeking care at either health facilities or shops. The intervention group was most likely to have their child seek care at school while the control group was most likely to seek private care. However, there was no difference between groups in relation to those reporting not seeking care from any source (figure 3).

### Surveillance for adverse events

Passive surveillance resulted in no reported serious adverse events (SAEs). However active surveillance revealed a total of 53 deaths during the 16-month study period (34 of 34 965 and 19 of 34 449 of the children enrolled in the intervention and control schools, respectively). In the intervention group, four of these deaths occurred within 30 days of seeking treatment from an LTK dispenser. In two cases, malaria was not implicated; in the third case the child tested positive by RDT, started on treatment and was referred to a health facility with danger signs; in the fourth case the child was recorded as RDT-negative after reporting to the LTK dispenser, but was subsequently taken to the health facility the next day where they were reported to be malaria-positive, and died the following week.

## Discussion

Despite a move towards increased integration of health interventions within the education sector there remains a lack of consistent evidence for such interventions in low-income countries, especially in relation to malaria.^42^ This cluster randomised trial, evaluating the impact of school-based malaria diagnosis and treatment using RDTs and ACTs on attendance, health and education, aimed to improve the evidence base and was demand-led by both the MoH and MoEST.

We did not detect an overall benefit of school-based diagnosis and treatment of malaria on school attendance, measured either by daily teacher-reported attendance or study officer spot check attendance, in this high transmission setting in Malawi. This finding is in contrast with previous school-based malaria studies^43^ including the PTK malaria case-management intervention implemented in Malawi using presumptive malaria treatment, which found a significant reduction in both general and illness-specific absenteeism.^22^ It is also contrary to the finding of MacNab *et al* 3 who observed a reduction in school absenteeism using a before-after design to evaluate a pilot programme in four schools in Uganda, in which teachers were trained to diagnose and treat uncomplicated malaria using RDTs and ACTs.^3^ However, neither of these studies were powered to detect a reduction in absenteeism, nor used a robust randomised study design.

It is worth mentioning that the lack of overall impact on school attendance reported in the current study is not uncommon in school-based health intervention trials^44 45^ and it is widely appreciated that both the measurement and attribution of absenteeism are challenging. This is due both to the multifactorial nature of absenteeism, and the resource-intensive process required to document individual-level attendance on a regular basis. While the findings of this study might have been strengthened by accounting for individual-level baseline characteristics and baseline measures of attendance, given the randomised nature of the study we have no reason to suspect that this differed across groups at baseline. The overall impact may have been masked by lack of effect for the children in the intervention group who did not directly benefit from the intervention, highlighting the increasingly cited issue of trial design for an intervention where a benefit is only required, sought or gained by a limited proportion of the intervention population.^46 47^ The nature of the intervention (treatment of symptomatic children) is such that there are no immediate benefits in terms of reduced transmission for the wider group, of one individual taking up the intervention, such as there might be with an intervention such as intermittent screening and treatment or intermittent preventive treatment.^18^ The increased absenteeism attributed to illness observed in the intervention group during spot checks was unexpected but could potentially be explained by factors related to the intervention design. Notably, an increased focus on illness in the intervention schools as a result of the study may have led to teachers reporting children as absent due to illness more frequently in the intervention schools rather than as absent for an unknown reason. In addition, children who had received treatment from the intervention may have been more likely to spend time recovering at home, in contrast to children in the control group, who would continue to attend school while still feeling unwell.

School attendance is known to be sensitive to contextual changes both at the school level, such as the introduction of a school feeding programme, or at the local environmental level, such as weather patterns and their effect on the local harvest.^6^ Such changes are unavoidable and though monitored and broadly accounted for wherever possible, they may have exerted unobserved heterogeneity on the primary outcome of attendance. Although measured at baseline and included in the initial study stratification, the extent of school-feeding programmes changed throughout the study period, and these are known to influence enrolment and attendance.^48–50^ Furthermore, information on whether the children were orphans or vulnerable children, or involved in child labour, both risk factors previously shown to be associated with attendance,^6 51^ were not collected. However, due to the randomised design, such changes and risk factors are likely to be similar across groups.

There was a surprisingly large reduction in *Plasmodium* prevalence (from approximately 50% to 14%) observed between the repeat cross-sectional surveys conducted at baseline, and those at follow-up (April–May 2011 and February–March 2015, respectively), and this was seen across both study groups. Further research is required into the impetus for this observed substantial decline. Walldorf *et al* reported an intermediate prevalence of 31% from 2012 to 2014 in neighbouring districts of Malawi^13^ and a national bednet distribution campaign was conducted following the baseline surveys in 2012. The absence of apparent differences between intervention and control groups in relation to health (*Plasmodium* infection or anaemia) could be attributed to the fact that the intervention involved the testing and treatment of symptomatic cases, and thus asymptomatic cases remained untreated. Furthermore, the unblinded nature of the trial may have led to biases such as a John Henry effect, with parents of children in the control group being more diligent in their treatment-seeking behaviour for their children, in light of the fact that they were unable to access the LTK intervention.

The lack of impact of the intervention on education outcomes is less surprising, given the lack of effect on health and absenteeism, with both sitting on the causal pathway for education outcomes. However, the results do indicate that concerns of a potential negative impact on children’s education in the intervention group due to the teachers’ class time being consumed by the LTK programme are unsupported. Nevertheless, the extent of teachers’ time used by the programme remains a concern in the rainy seasons, when the number of LTK consultations was high, and would need to be addressed before any future implementation.

Despite persistent global gender inequalities in healthcare, analysis of the LTK treatment records revealed that a significantly greater proportion of consultations were with female children, even in a setting of relative gender parity by school enrolment. Given that school-based case management would be expected to remove many of the economic and social barriers responsible for such inequalities, this disparity warrants further investigation. This pattern of usage suggests that the LTK may be an effective way of reaching this group with additional healthcare interventions such as menstrual hygiene management and adolescent nutrition. While several school-based health interventions have resulted in a greater impact on female students, especially in the domain of school enrolment and attendance,^49 52^ despite the greater uptake of LTK intervention among girls, no differential impact was observed by sex for any of the outcomes.

Further possible reasons for the lack of effect may be raised by examination of the causal chain of impact, with the key stages identified as children’s health-seeking behaviour, correct diagnosis and management by the LTK dispenser and ACT compliance. If the high LTK consultation numbers were due to replacing health-sector and private-sector services with the school-based service—a substitution effect—rather than an increase in care seeking overall in the intervention group, this may have meant no overall increase in children treated. In which case, the intervention may not be expected to have an impact on health and absenteeism. A further factor is that children in the intervention schools, once diagnosed and treated, may have been sent home for the day and subsequently remained at home during the 3-day course of treatment to recover. Paradoxically, this confirmed malaria diagnosis may have encouraged a greater degree of absenteeism than in those who continue to feel ill but are not able to seek treatment easily so continue to attend school. Furthermore, compliance to the ACT - artemether lumefantrine (AL) - was not confirmed across the six AL doses as only the first dose was being observed (as in health facilities). Evidence from 2009 reported that compliance with AL as part of routine clinical care for Malawian school-age children (5–17 years of age) was moderate (60.6%).^53^ In future, a rigorous process evaluation, monitoring key implementation indicators would be valuable for examining such issues in greater depth.

The results regarding the uptake of the intervention suggests the LTK programme provided a valued and well-used service, a finding corroborated by qualitative discussions and interviews with teachers, community members and policy makers alike.^54^ As previously reported in evaluations of community-based case management of malaria, studies have frequently observed high uptake of such services in a range of settings,^55 56^ and sudden and significant declines in malarial care-seeking visits following the introduction of CHW-delivered community-based case management of malaria.^57^ Despite a shift in care-seeking away from public health facilities reported by parents in intervention schools, we were unable to further investigate this at the health centre level. In light of serious constraints to the health system in Malawi, this is worthy of further investigation given the high and sustained uptake of the intervention and potential to shift workload away from already strained health centres.

## Conclusions

This trial found no overall impact of the school-based malaria case management programme on either school absenteeism, health or education outcomes. Despite this, both the rate of uptake of the intervention and the results from a qualitative evaluation^54^ demonstrated that the LTK programme was in high demand and well perceived. Future work is required to explore how the programme could be integrated alongside or supplement routine community and facility-based healthcare services to ensure universal access to malaria diagnosis and treatment. The success of the intervention in terms of providing such a well-used service, particularly by girls, also indicates a role for the LTK as a platform for providing additional services targeted at this group (eg, menstrual hygiene management and sexual and reproductive health).
