# Supplementary material for: Impact of school-based malaria case management on school attendance, health and education outcomes: a cluster randomised trial in southern Malawi
Source: BMJ Glob Health. 2020 Jan 14;5(1):e001666. doi: 10.1136/bmjgh-2019-001666 (PMC7042571; doi:10.1136/bmjgh-2019-001666)
Supplement: Supplementary data [file bmjgh-2019-001666supp001.pdf]

Supplementary File: Checklist S1: CONSORT 2010 checklist

Section/Topic	Item No	Standard Checklist item	Extension for cluster designs	Page No *
**Title and abstract**				
	1a	Identification as a randomised trial in the title	Identification as a cluster randomised trial in the title	Title page (1) – stated in title
	1b	Structured summary of trial design, methods, results, and conclusions (for specific guidance see CONSORT for abstracts)[i,ii]	See table 2	Abstract, lines 29 -57
**Introduction**				
**Background and objectives**	2a	Scientific background and explanation of rationale	Rationale for using a cluster design	Introduction, paragraph 3
	2b	Specific objectives or hypotheses	Whether objectives pertain to the the cluster level, the individual participant level or both	Methods, "Study design" paragraph 4
**Methods**				
**Trial design**	3a	Description of trial design (such as parallel, factorial) including allocation ratio	Definition of cluster and description of how the design features apply to the clusters	Methods, "Study design" paragraph 4 & "Intervention" 7-8
	3b	Important changes to methods after trial commencement (such as eligibility criteria), with reasons		N/A
**Participants**	4a	Eligibility criteria for participants	Eligibility criteria for clusters	Methods, "Sample size and randomisation" paragraph 6
	4b	Settings and locations where the data were collected		Methods, "Study Setting" paragraphs 2 & 3
**Interventions**	5	The interventions for each group with sufficient details to allow replication, including how and when they were actually administered	Whether interventions pertain to the cluster level, the individual participant level or both	Methods, "Intervention" paragraphs 4 & 7-9
**Outcomes**	6a	Completely defined pre-specified primary and secondary outcome measures, including how and when they were assessed	Whether outcome measures pertain to the cluster level, the individual participant level or both	Methods, "Assessments" paragraphs 10-14
	6b	Any changes to trial outcomes after the trial commenced, with reasons		N/A
**Sample size**	7a	How sample size was determined	Method of calculation, number of clusters(s) (and whether equal or unequal cluster sizes are assumed), cluster size, a coefficient of intracluster correlation (ICC or *k*), and an indication of its uncertainty	Methods, "Sample size and randomisation" paragraphs 5-6
	7b	When applicable, explanation of any interim		N/A

		analyses and stopping guidelines		
**Randomisation:**				
**Sequence generation**	8a	Method used to generate the random allocation sequence		Methods, "Sample size and randomisation" paragraph 6
	8b	Type of randomisation; details of any restriction (such as blocking and block size)	Details of stratification or matching if used	Methods, "Sample size and randomisation" paragraph 6
**Allocation concealment mechanism**	9	Mechanism used to implement the random allocation sequence (such as sequentially numbered containers), describing any steps taken to conceal the sequence until interventions were assigned	Specification that allocation was based on clusters rather than individuals and whether allocation concealment (if any) was at the cluster level, the individual participant level or both	Methods, "Study design" paragraph 4
**Implementation**	10	Who generated the random allocation sequence, who enrolled participants, and who assigned participants to interventions	Replace by 10a, 10b and 10c	
	10a		Who generated the random allocation sequence, who enrolled clusters, and who assigned clusters to interventions	Methods, "Sample size and randomisation" paragraph 6
	10b		Mechanism by which individual participants were included in clusters for the purposes of the trial (such as complete enumeration, random sampling)	Methods, "Sample size and randomisation" paragraph 6
	10c		From whom consent was sought (representatives of the cluster, or individual cluster members, or both), and whether consent was sought before or after randomisation	Methods, "Ethics statement" paragraph 18
**Blinding**	11a	If done, who was blinded after assignment to interventions (for example, participants, care providers, those assessing outcomes) and how		Methods, "Study design" paragraph 4
	11b	If relevant, description of the similarity of interventions		Methods, "Study design" paragraph 4
**Statistical methods**	12a	Statistical methods used to compare groups for primary and secondary outcomes	How clustering was taken into account	Methods, "Data analysis" paragraphs 16-17
	12b	Methods for additional analyses, such as		Methods, "Data analysis" paragraph 17

		subgroup analyses and adjusted analyses		
**Results**				
**Participant flow (a diagram is strongly recommended)**	13a	For each group, the numbers of participants who were randomly assigned, received intended treatment, and were analysed for the primary outcome	For each group, the numbers of clusters that were randomly assigned, received intended treatment, and were analysed for the primary outcome	Results, "Baseline characteristics and trial profile" paragraphs 2-3 and Figure 2
	13b	For each group, losses and exclusions after randomisation, together with reasons	For each group, losses and exclusions for both clusters and individual cluster members	Results, "Baseline characteristics and trial profile" paragraphs 2-3 and Figure 2
**Recruitment**	14a	Dates defining the periods of recruitment and follow-up		Methods, "Data analysis" paragraph 17 and Figure 1
	14b	Why the trial ended or was stopped		Methods, "Intervention" paragraph 8
**Baseline data**	15	A table showing baseline demographic and clinical characteristics for each group	Baseline characteristics for the individual and cluster levels as applicable for each group	Results, "Baseline characteristics and trial profile" paragraph 1 and Table 1
**Numbers analysed**	16	For each group, number of participants (denominator) included in each analysis and whether the analysis was by original assigned groups	For each group, number of clusters included in each analysis	Results, "Baseline characteristics and trial profile" paragraphs 2-3 and Figure 2
**Outcomes and estimation**	17a	For each primary and secondary outcome, results for each group, and the estimated effect size and its precision (such as 95% confidence interval)	Results at the individual or cluster level as applicable and a coefficient of intracluster correlation (ICC or k) for each primary outcome	Supplementary Table S2
	17b	For binary outcomes, presentation of both absolute and relative effect sizes is recommended		Tables 4-6
**Ancillary analyses**	18	Results of any other analyses performed, including subgroup analyses and adjusted analyses, distinguishing pre-specified from exploratory		Table 2-3 Supplementary table S1
**Harms**	19	All important harms or unintended effects in each group (for specific guidance see CONSORT for harms[iii])		Results, "Surveillance for adverse events" paragraph 13
**Discussion**				
**Limitations**	20	Trial limitations, addressing sources of potential bias, imprecision, and, if relevant, multiplicity of analyses		Discussion, paragraphs 3-5

**Generalisability**	21	Generalisability (external validity, applicability) of the trial findings	Generalisability to clusters and/or individual participants (as relevant)	Discussion, paragraphs 1 & 9 and "Conclusions"
**Interpretation**	22	Interpretation consistent with results, balancing benefits and harms, and considering other relevant evidence		Discussion, paragraphs 2, 3 and 5
**Other information**				
**Registration**	23	Registration number and name of trial registry		Methods, "Ethics statement" paragraph 18 and under abstract
**Protocol**	24	Where the full trial protocol can be accessed, if available		Supplementary File S2
**Funding**	25	Sources of funding and other support (such as supply of drugs), role of funders		"Funding" subheading

** Note: page numbers optional depending on journal requirements*

Table 2: Extension of CONSORT for abstracts[i,ii]

Item	Standard Checklist item	Extension for cluster trials	
**Title**	Identification of study as randomised	Identification of study as cluster randomised	Explicitly stated in title.
**Trial design**	Description of the trial design (e.g. parallel, cluster, non-inferiority)		Stated in title and abstract
**Methods**			
**Participants**	Eligibility criteria for participants and the settings where the data were collected	Eligibility criteria for clusters	Participant eligibility in the manuscript body text. Data collection settings also included in abstract - methods
**Interventions**	Interventions intended for each group		Included in abstract
**Objective**	Specific objective or hypothesis	Whether objective or hypothesis pertains to the cluster level, the individual participant level or both	Included in abstract – methods
**Outcome**	Clearly defined primary outcome for this report	Whether the primary outcome pertains to the cluster level, the individual participant level or both	Included in abstract – methods
**Randomization**	How participants were allocated to interventions	How clusters were allocated to interventions	Although not stated explicitly in the abstract, these details are provided in the manuscript body text.
**Blinding (masking)**	Whether or not participants, care givers, and those assessing the outcomes were blinded to group assignment		Although not stated explicitly in the abstract, these details are provided in the manuscript body text.
**Results**			
**Numbers randomized**	Number of participants randomized to each group	Number of clusters randomized to each group	Included in abstract – methods
**Recruitment**	Trial status[1]		
**Numbers analysed**	Number of participants analysed in each group	Number of clusters analysed in each group	Though not stated explicitly in the abstract, these details are provided in the manuscript body text and Figure 2
**Outcome**	For the primary outcome, a result for each group and the estimated effect size and its precision	Results at the cluster or individual participant level as applicable for each primary outcome	Included in abstract – results
**Harms**	Important adverse events or side effects		Although not stated explicitly in the abstract, these details are provided in the manuscript body text.
**Conclusions**	General interpretation of the results		Included in abstract – conclusion
**Trial registration**	Registration number and name of trial register		End of abstract and in ethics statement in body in manuscript body text
**Funding**	Source of funding		A funding statement is provided.

REFERENCES

i Hopewell S, Clarke M, Moher D, Wager E, Middleton P, Altman DG, et al. CONSORT for reporting randomised trials in journal and conference abstracts. *Lancet* 2008, 371:281-283

ii Hopewell S, Clarke M, Moher D, Wager E, Middleton P, Altman DG at al (2008) CONSORT for reporting randomized controlled trials in journal and conference abstracts: explanation and elaboration. *PLoS Med* 5(1): e20

iii Ioannidis JP, Evans SJ, Gotzsche PC, O'Neill RT, Altman DG, Schulz K, Moher D. Better reporting of harms in randomized trials: an extension of the CONSORT statement. *Ann Intern Med* 2004; 141(10):781-788.

[1] Relevant to Conference Abstracts
