# Supplementary material for: Impact of school-based malaria case management on school attendance, health and education outcomes: a cluster randomised trial in southern Malawi
Source: BMJ Glob Health. 2020 Jan 14;5(1):e001666. doi: 10.1136/bmjgh-2019-001666 (PMC7042571; doi:10.1136/bmjgh-2019-001666)
Supplement: Supplementary data [file bmjgh-2019-001666supp002.pdf]

Protocol S1

ADDITIONAL FILE 1 – STUDY PROTOCOL

Title of project

Feasibility and impact of a school-based malaria control intervention on health and education in Malawian schoolchildren

1. Investigators and Institutional Affiliations

Principal investigators:
Dr Don Mathanga (MBBS, PhD)[1]
Professor Simon Brooker (MSc, PhD)[2,3]

Co-investigators:
John Sande (MSc)[4]
Reuben Mwenda (MSc)[5]
Natalie Roschnik (MPH)[6]
Charles Mazinga (MSc)[7]
Katherine Halliday (MSc)[2]
Joby George (MSc)[8]

[1]College of Medicine, Malaria Alert Centre, Blantyre, Malawi
[2]London School of Hygiene and Tropical Medicine, London, UK
[3]KEMRI-Wellcome Trust Research Programme, Kenya
[4]National Malaria Control Program, Ministry of Health, Malawi
[5]Health Technical Support Services-Diagnostics , Ministry of Health, Malawi
[6]Save the Children, USA
[7]School Health and Nutrition, Ministry of Education, Malawi
[8]Save the Children, Malawi

See Appendix 1 for CVs of Investigators

Halliday KE, *et al. BMJ Global Health* 2020; 5:e001666. doi: 10.1136/bmjgh-2019-001666

LIST OF ACRONYMS

**ACT**	Artemesinin Combination Therapy
**DHO**	District Health Officer
**DEM**	District Education Manager
**DHS**	Demographic and health survey
**IPT**	Intermittent preventive treatment
**IST**	Intermittent Screening and Treatment
**ITN**	Insecticide-treated mosquito net
**KEMRI**	Kenya Medical Research Institute
**LA**	Lumefantine and Artemether
**LLIN**	Long-lasting insecticidal net
**LSHTM**	London School of Hygiene and Tropical Medicine
**MAC**	Malaria Alter Center
**MDG**	Millennium Development Goals
**MOEST**	Ministry of Education Science and Technology
**MOH**	Ministry of Health
**NMCP**	National Malaria Control Program
**NMSP**	National Malaria Strategic Plan
**NSO**	National Statistics Office
**PEA**	Primary Education Advisor
**PI**	Principal Investigator
**PTA**	Parent Teacher Association
**PTK**	Pupil Treatment Kit
**RDT**	Rapid diagnostic test
**SHN**	School Health and Nutrition
**SHP**	School Health Program
**SMC**	School Management Committee
**SP**	Sulphadoxine-pyrimethamine
**TA**	Traditional Authority
**UNICEF**	United Nation's Children Fund
**USAID**	United States Agency for International Development
**WHO**	World Health Organization

Halliday KE, *et al. BMJ Global Health* 2020; 5:e001666. doi: 10.1136/bmjgh-2019-001666

EXECUTIVE SUMMARY

Both the health and education sectors are increasingly recognizing the importance of reducing incidences of malaria in school children. Although all-cause mortality rates are lowest among school-age children, it is estimated that malaria causes up to 50% of all deaths in this age group in Africa. From an education perspective, malaria contributes between 5% to 8% of all-cause absenteeism among African school children, equivalent to 50% of all preventable absenteeism or 4 to 10 million school days lost per year. The Government of Malawi is committed to improving the health and education of its children, and recognizes the importance of child health for educational achievement. Schools and teachers can and should play a key role in malaria prevention. The Malawi Malaria Strategic Plan 2011-2015: Towards Universal Access emphasizes, in Objective 1, the need for effective and prompt diagnosis and treatment. However, the evidence base for policy development and program implementation for school-based malaria control remains inadequate.

In 2007, the Ministry of Education launched a national School Health and Nutrition (SHN) program with the inclusion of first aid kits called Pupil Treatment Kits (PTKs). Unfortunately, when the government changed the first line malaria treatment from Sulphadoxine-pyrimethamine (SP) to Artemisinin Combination Therapy (ACT) the PTKs were withdrawn. The introduction of Rapid Diagnostic Tests (RDTs) to diagnose *P. falciparum* infection (required before treating with ACT) provides a new opportunity to scale-up simple and cost-effective malaria diagnosis by teachers through RDTs and treatment with ACT. Therefore the overall study objective is to evaluate the feasibility, acceptability, impact and cost effectiveness of school-based RDT diagnosis and ACT treatment of uncomplicated malaria cases in schoolchildren**,** with specific objectives evaluating the effectiveness of the school-based diagnosis and treatment of uncomplicated malaria in 1) reducing children's absenteeism from school; 2) improving educational performance; and 3) reducing morbidity.

The study will be conducted by Save the Children in collaboration with the National Malaria Control Programme (Ministry of Health), Health Technical Support Services-Diagnostics (Ministry of Health), Malaria Alert Centre, London School of Hygiene and Tropical Medicine, KEMRI-Wellcome Trust Research Centre and the national School Health and Nutrition Program (Ministry of Education, Science and Technology). This study will be a cluster-randomised controlled trial with the target population in this study including children attending primary schools in Malawi. The accessible population for this intervention includes the children attending the 58 participating primary schools in standards 1-8 in Traditional Authority (TA) Chikowi, Zomba District. Inclusion criteria includes: 1) Pupil enrolled at participating schools in standards 2, 4 and 6; 2) Provision of informed consent from parent or guardian; 3) Provision of assent by student. Exclusion criteria include pupils unwilling to participate in the study and pupils known to have a chronic medical condition, which will affect their school attendance in the long term.

Schools will be randomized to one of two groups: 1) receiving either the PTKs (first aid kits) including RDT and ACTs for diagnosis and treatment of uncomplicated malaria in addition to teacher training for the use of these items; or 2) the counterfactual. The attendance, health and education outcomes will be assessed at the end of the intervention period. Data collected as part of a needs assessment for malaria control in 50 schools in TA Chikowi in May 2011 will provide baseline measurements of *P.falciparum* infection, symptomatic malaria, anaemia and educational performance. Data collected as part of Save the Children's ongoing Basic Education program in TA Chikowi will provide baseline data on educational indicators and school attendance. A total sample of 60 children (20 from each standard 2, 4, and 6) from 29

schools equates to 1740 children in each group will be used. Therefore, a total of 58 schools and 3480 children will be involved in the study.

Treatment schools will receive PTKs including RDTs and ACT, and training for two teachers from each school in the use of the items in the PTKs. Teachers will be assessed in their ability to carry out the procedure safely and accurately. Surveys include: teacher information, parent questionnaire, and child health questionnaire. Attendance will be monitored through class registers and spot checks. Qualitative data collection will take place through focus group discussions with key stakeholders. The feasibility of training teachers in the use of the PTKs will be measured via a series of random supervisory spot checks made by a health worker to assess teachers' ability to correctly use the logarithms, RDTs, and provide treatment to children in a professional manner.

There are minimal risks involved in this study. The collection of finger-prick blood samples may lead to transient bleeding or minor pain for children, however, precautions will be taken to avoid bleeding and risks of infection will be minimized. The study involves specific risks from the ACT drugs but ACTs are remarkably well tolerated in humans, with no serious adverse events or significant toxicity reported. While the risks are minimal the benefits are great. If a relationship demonstrates that malaria treatment in schools improves school attendance and other health and educational outcomes, and is feasible and cost effective, the NMCP and national SHN program are keen to scale this intervention up to all schools in Malawi. Additionally, children randomized to the PTK intervention arm will benefit from free medical treatment provided by the trained teachers.

No participant will receive compensation for their involvement in the intervention except the teachers for whom participation costs for the training workshop are covered. All records will be confidential; participants will be identified primarily by their study number and patient names will not be entered into the computerized database. No individual identities will be used in any reports or publications resulting from the study.

The results will be disseminated primarily to the NMCP (Ministry of Health), the SHN Program (Ministry of Education, Science and Technology), the District Health and District Education Offices, and other interested stakeholders at national and local levels. It is also anticipated that the results will be presented at appropriate national and international scientific meetings and several papers will be written and submitted to peer-reviewed scientific journals for publication. This study will contribute to the global evidence base on malaria control in schools and inform malaria control in schools strategies in other countries in the region.

I. BACKGROUND

Burden of malaria among school children

Both the health and education sectors are increasingly recognizing the importance of malaria for school children (Brooker, Kolaczinski et al. 2009). Although all-cause mortality rates are lowest among school-age children, it is estimated that malaria causes up to 50% of all deaths in this age group in Africa (Snow et al., 2003). Among children attending school, studies suggest that between 20% to 50% of school children living in areas of stable transmission experience clinical malaria attacks each year (Clarke, Brooker et al. 2004). School children are also prone to harbor non-symptomatic malaria infection: in Malawi, for example, up to 50% of school children are infected with *Plasmodium* infection (PMI, 2009). Malaria (both clinical and asymptomatic malaria) is a major cause of anemia among school-age children and efforts to control malaria among this age group can dramatically improve hemoglobin levels (Geerligs, Brabin et al. 2003).

From an education perspective, malaria contributes between 5% to 8% of all-cause absenteeism among African school children, equivalent to 50% of all preventable absenteeism and 4 to 10 million school days lost per year (Brooker, Guyatt et al. 2000). In addition, it is documented that malaria, particularly cerebral malaria, can cause impairment that negatively affects intellectual development in children (Fernando, De Silva et al. 2003). Recent evidence from Kenya suggests that non-severe malaria can adversely affect cognition and attention, but that preventing malaria, through intermittent preventive treatment (IPT) - the mass administration of a full therapeutic course of an anti-malarial drug irrespective of infection status - can cost-effectively reverse these educational impacts (Clarke, Jukes et al. 2008; Temperley, Mueller et al. 2008).

Malaria in Malawi

Malaria is a major public health problem in Malawi, estimated to be endemic in 95% of the country with over 85% of malaria infections due to *Plasmodium falciparum* (PMI, 2009). Transmission is perennial in most of the country, with seasonal peaks during the rainy season, roughly from December through March and reduced but persistent clinical disease, and presumably transmission, throughout the dry season. In April 2010, Save the Children, Malaria Alert Center and the LSHTM conducted a needs assessment in 50 schools targeted by the study in TA Chikowi and found the prevalence of *Plasmodium falciparum* and anaemia to be 60.0% and 32.4%, respectively (unpublished data). In 2003, data collected by Save the Children in another study showed that malaria was the leading cause of death for school age children (Pasha, Del Rosso et al. 2003). School-aged children in Malawi are estimated to experience 0.59 clinical attacks of malaria each year, equivalent to 2.1 million attacks among Malawian school-aged children (Muula, Rudatsikira et al. 2007). Such health problems experienced by Malawian schoolchildren are likely to have an impact on their school attendance, learning and education.

Save the Children in Malawi

Save the Children began operating in Malawi in 1983, and its main goal has been to improve children's health and educational status, and building community and partner capacity to sustain those improvements. Save the Children has been the leading NGO working with the Ministry of Health to scale up the national maternal, newborn and child health package, including Community Management of malaria. Save the Children also implemented a School Health and Nutrition (SHN) program for more than 10 years in partnership with the district health and education authorities in Mangochi and Balaka districts. Starting in 2000, Save the Children trained teachers to treat malaria in schools using a Pupil Treatment Kit (PTK)

including Sulphadoxine-Pyrimethamine (SP). In each school, three teachers were trained to recognize the signs and symptoms used to diagnose malaria and safely administer the antimalarial treatment; a simple algorithm for diagnosis was created for use by teachers. Sick children were reported to teachers and suspected malaria cases were treated with SP according to the national guidelines where antipyretics were provided to the sick children to take home; nearly 11,000 children in 101 schools were treated for malaria over the course of a year. Sick children whose health did not improve were referred to a health facility. The overall and malaria-specific mortality rates for the 3 years before and 2 years after the intervention dropped from 2.2 to 1.44 deaths/1000 student-years and from 1.28 to 0.44 deaths/1000 student-years, respectively (Pasha, Del Rosso et al. 2003). A retrospective analysis showed that the program reduced school absenteeism and grade repetition (Simwaka, Simwaka et al. 2009). Focus group discussions with students in Balaka District suggested that the average number of days of schooling missed by students with malaria fell from seven to just two days when early treatment became available at schools.

Options for school-based malaria control

To help improve the health and education of school children, governments are increasingly implementing school health programs, and are already providing them with health education and health services such as deworming and micronutrient supplementation. These interventions are simple, safe and familiar, and address problems that are widespread and recognized as important within the community. This existing infrastructure could also deliver school-based malaria interventions, but there is currently no consensus as to the optimal approach. Malaria prevention education in schools focusing prompt diagnosis and treatment of malaria and use of Long-lasting insecticidal nets (LLINs) by all family members are essential in all settings and will help improve behaviors in the community. Other school-based strategies include intermittent screening and treatment (IST) and intermittent preventive treatment (IPT) which involves treating both symptomatic and asymptomatic children at regular intervals throughout the school year to clear them of parasites and reduce their risk of becoming anemic, which in turn affects their ability to concentrate and learn in school. IPT involves treating all children regardless of infection status and IST involves screening children first using Rapid Diagnostic Tests (RDTs) and only treating those that are positive. Unfortunately neither of these are considered viable options for Malawi, because of concerns over drug resistance, and because transmission is high all year round which means children are likely to become re-infected shortly after the treatment, reducing its' cost effectiveness.

In June 2012, a national stakeholders hosted by the National Malaria Control Program (NMCP) presented the results of the 2011 needs assessment in schools and recommended re-visiting teacher based treatment of symptomatic cases in schools, this time using RDTs and ACTs. This would support the NMCP's new 2011-2015 strategic plan which aims to achieve universal coverage in the prevention and treatment of malaria. Their goal is to reduce by half the 2010 levels of malaria morbidity and mortality in Malawi by the year 2015 by ensuring that every citizen (including school age children who represent nearly a third of the population) are reached with malaria interventions including care and effective cure (MoH 2011). From an education point of view, school based malaria treatment would contribute to the School Health and Nutrition 2008-2018 strategic plan which aims to have healthy school-age children who can fulfill their optimum learning potential, by providing school-based health and nutrition services as an integral, sustainable part of the education system.

Scale up of malaria control efforts using insecticide treated mosquito nets (ITNs) and indoor residual spraying (IRS) has led to a decrease in malaria transmission and subsequent decline

in the proportion of fevers attributable to malaria. These factors have increased the importance of obtaining accurate malaria diagnosis based on the demonstration of parasitaemia prior to treatment to ensure that malaria treatment is only targeted to patients who need them. In line with the World Health Organization (WHO) recommendations and the Malawi Malaria Strategic Plan (MSP) 2011–2015, the Malawi National Malaria Control Program (NMCP) developed new malaria treatment algorithms, which incorporate the use of rapid diagnostic tests (RDTs) for definitive diagnosis of malaria in patients aged ≥5 years who seek care in health facilities and where malaria microscopy is not available (MoH 2011). This could be extended to schools by training teachers to safely and accurately perform rapid diagnostic tests to provide a parasitologically confirmed diagnosis of uncomplicated malaria; and based on the results treat with ACT or refer to the health facility. This school-based intervention will ensure prompt access to diagnosis and treatment for school-going children.

Teacher delivery of health services has been successfully implemented for other school-based programmes such as deworming. In Ghana, Tanzania and Uganda, most teachers delivering drugs as part of large-scale programmes were positive about their role when interviewed. Parents in Ghana and Tanzania were accepting of teachers delivering the drugs (Brooker, Marriot et al. 2001; Fleming, Fenwick et al. 2009). A recent study in Kenya highlighted concerns by both parents and teachers about teachers safely handling blood and sharps and their ability to arrive at the correct test result (Okello, Ndegwa et al. 2012). However, focus group discussions found that community members and health workers supported the idea of teachers using RDTs provided they are given thorough practical training to acquire additional skills, accurate information, and guidelines to ensure safety, and are mentored by health workers.

Previous studies in Ghana, Malawi and Tanzania have found that teachers can effectively deliver malaria treatment (Magnussen, Ndawi et al. 2001; Pasha, Del Rosso et al. 2003; Afenyadu, Agyepong et al. 2005). However, to date no studies investigated the operational feasibility of using teachers to parasitologically diagnose uncomplicated malaria and treat based on the results. WHO researchers in Zambia have developed a Job Aid (set of clear pictorial instructions for routine use) and a corresponding training programme (Harvey, Jennings et al. 2008). Such training aids may also be used to train teachers to conduct RDTs in schools. The use of RDTs by non-medical personnel has been successfully trialled in a number of settings (Drummond 2005; Elmardi, Malik et al. 2009; Hawkes, Katsuva et al. 2009; Lemma, Byass et al. 2010). Kyabayinze and Asiimwe conducted one-day training on the use of RDTs to health workers, some with formal medical training and some without, and found that training successfully provided adequate skills and competency with RDTs. Additionally, they found that the use of RDTs by both the medical and non-medical workers were acceptable and feasible, provided clear guidelines exist (Asiimwe, Kyabayinze et al. 2012; Kyabayinze, Asiimwe et al. 2012). Currently in Malawi, the Ministry of Health is conducting an operational study to assess how a package for diagnosis of malaria with RDTs and treatment of uncomplicated malaria with ACTs in patients of all ages can best be delivered at the community level by government paid community health workers (Health Surveillance Assistants). In the current policy climate of targeting treatment to parasitologically confirmed cases, a study to investigate the use of RDTs by teachers is a logical next step.

II. JUSTIFICATION

The Government of Malawi is committed to improving the health and education of its children, and recognizes the importance of child health for educational achievement. Schools

and teachers can and should play a key role in malaria prevention. The Malawi Malaria Strategic Plan 2011-2015 Towards Universal Access emphasizes in Objective 1 the need of effective and prompt diagnosis and treatment (MoH 2011). Schools and teachers can act as an efficient delivery point for universal access for school-age children given the high school enrollment in Malawi (92.8%) (UNICEF 2008). However, the evidence base for policy development and program implementation for school-based malaria control remains inadequate.

In 2007, the Ministry of Education launched a national SHN program. One of the key elements of this program was the inclusion of PTKs, piloted by Save the Children in Mangochi district, which were scaled up to all schools across the country. Unfortunately, when the government changed the first line malaria treatment from SP to ACTs the PTKs were withdrawn. This is because regulation states that in order to treat with ACT in children over the age of five years a clinical diagnosis (via RDT or microscopy) must be made first. However, with the national introduction of RDTs to diagnose *Plasmodium falciparum* infection in Malawi and the subsequent roll-out in health facilities, and with teachers being at the frontline for providing health interventions for school-age children, there is a need for evaluating the feasibility and cost effectiveness of engaging school teachers to diagnose malaria using RDTs and to provide treatment using ACTs. This approach is a potentially valuable intervention in schools for improving both health and education goals. The delivery by teachers makes it affordable and cost-effective, thereby increasing the scalability potential for Malawi.

The findings of this activity will help inform policy makers on how to proceed with school-based malaria control planning in Malawi, especially in moving closer to the goal of universal access to prompt diagnosis and treatment. The cost effectiveness for improving both health and educational outcomes and the potential for scale up of such an intervention at National level is of great interest to policy makers. A particular strength of the study is the strong policy linkages with both health and education sectors in Malawi. These sectors are seeking clear policy and technical guidance for the optimal approach to malaria control in schools.

III. NULL HYPOTHESIS

School-based malaria control utilizing teachers for diagnosis (with RDTs) and treatment (with ACTs), provided alongside other treatments for common health problems (Pupil Treatment Kits - PTKs) will not reduce school absenteeism and child morbidity.

IV. OBJECTIVES

a. Overall Objective:

To evaluate the feasibility, acceptability, impact and cost effectiveness of school-based RDT diagnosis and treatment of uncomplicated malaria cases, provided as part of a PTK, in schoolchildren**.**

b. Specific objectives:

i. To evaluate the effectiveness of the school-based diagnosis and treatment of uncomplicated malaria in reducing children’s absenteeism from school.

ii. To evaluate the effectiveness of the school-based diagnosis and treatment of uncomplicated malaria in improving educational performance.

iii. To evaluate the effectiveness of the school-based diagnosis and treatment of uncomplicated malaria in improving the daily health status (reducing morbidity) of schoolchildren.

iv. To evaluate the impact of the school-based diagnosis and treatment of uncomplicated malaria on treatment seeking behavior at health facilities.

v. To evaluate the feasibility of using teachers to diagnose and treat uncomplicated malaria in schools using rapid diagnostic tests and to measure the retention of the skills and knowledge acquired following the training.

vi. To determine the cost and cost effectiveness of the school-based diagnosis and treatment of uncomplicated malaria

vii. To assess the acceptability of school-based diagnosis and treatment of uncomplicated malaria by parents, teachers and health care providers.

V. DESIGN AND METHODOLOGY

i) General Study design

This study will be a cluster-randomised controlled trial, to assess the feasibility and effectiveness of school-based diagnosis and treatment of uncomplicated malaria by teachers in reducing absenteeism and morbidity in schoolchildren.

The target population in this study includes children attending primary schools in Malawi. The accessible population for this intervention includes the children attending the 58 participating primary schools in standards 1-8 in Traditional Authority (TA) Chikowi, Zomba District. The unit of analysis is the school. Schools will be randomized to one of two groups, receiving either the Pupil Treatment Kits (first aid kits) including RDT and ACTs for diagnosis and treatment of uncomplicated malaria in addition to teacher training for the use of these items or the counterfactual. The attendance, health and education outcomes will be assessed at the end of the intervention period. Data collected as part of a needs assessment for malaria control in 50 schools in TA Chikowi in May 2011 will provide baseline measurements of *P.falciparum* infection, symptomatic malaria, anaemia and educational performance. Data collected as part of Save the Children's ongoing Basic Education program in TA Chikowi, will provide baseline data on educational indicators and school attendance. The intervention will begin in October 2012 and will end in June 2013.

The study will be conducted by Save the Children in collaboration with the National Malaria Control Programme (MoH), Health Technical Support Services-Diagnostics (MOH), Malaria Alert Centre, London School of Hygiene and Tropical Medicine, KEMRI-Wellcome Trust Research Centre and the national School Health and Nutrition Program (Ministry of Education, Science and Technology).

Halliday KE, *et al. BMJ Global Health* 2020; 5:e001666. doi: 10.1136/bmjgh-2019-001666

ii) Outcome measures

Primary outcome:

1. School attendance assessed through school attendance registers and random spot checks

Secondary outcomes:

2. Child morbidity as assessed by child reported daily health status charts
3. Educational performance as assessed by classroom-based tests of attention, literacy and numeracy
4. *P. Falciparum* parasitaemia and hemoglobin levels as assessed by health surveys conducted at the 9 month follow up
5. Treatment seeking at health facilities as assessed by child health record cards
6. Skills and knowledge retained by teacher as assessed by a questionnaire and practical assessments
7. Costs of the PTKs over the study period
8. Acceptability of teacher-led RDTs as assessed by focus group discussions with parents, children and community members
9. Frequency of use of school based treatment by children as assessed by PTK records

iii) Study site

The study site is TA Chikowi, Zomba District, located in the Southern part of Malawi, South of Lake Malawi. The TA has 438 villages with a population of 156,576. The area encompasses 7 zones and 58 schools with a total enrolment of 60 824, including 30 137 boys and 30 691 girls. School attendance in the area is erratic as seen from the average daily attendance, which is 66% for boys and 72% for girls. TA Chikowi has the highest enrolment in the district and a teacher pupil ratio of 1: 125. Malaria is hyperendemic with seasonal peaks during the rainy season, December to April. Malaria transmission throughout the district is high, with up to 57% of individuals infected with *P. falciparum* (Kazembe, Kleinschmidt et al. 2006). The needs assessment conducted in April 2010 among school children in 50 schools found that the prevalence of malaria infection among schoolchildren in TA Chikowi was 60% (ranging from 32-84% by school), and 32% (5-73% by school) of children were anaemic (unpublished data).

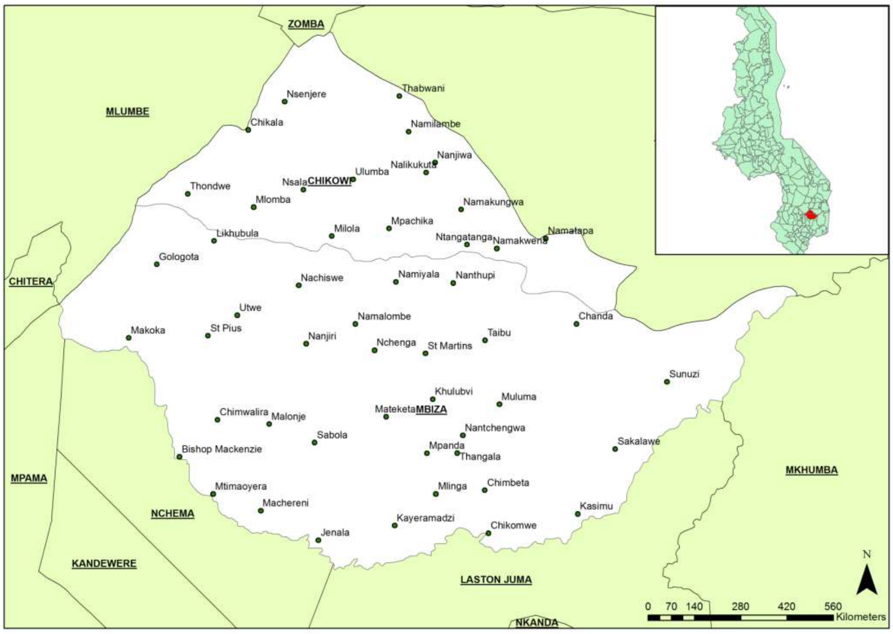

Fig 1. A map of the 50 schools surveyed during the needs assessment in 2011 which will serve as the baseline data for this study. An additional 8 schools will be included for the impact evaluation

iv) Study populations

All children (ECD and classes 1-8) attending the schools selected to receive the intervention will have access to the PTK, including the malaria treatment, when and if they report to the teacher as feeling unwell. The use of this service is therefore entirely voluntary.

For the evaluation survey, children enrolled in participating schools will be assessed for the following eligibility criteria to be included in the survey:

Inclusion criteria
- Pupil enrolled at participating schools in standards 2, 4 and 6
- Provision of informed consent from parent or guardian
- Provision of assent by student

Exclusion criteria
- Pupils unwilling to participate in the study
- Pupils known to have a chronic medical condition, which will affect their school attendance in the long term.

v) Sample size determination

This is an evaluation of an intervention implemented by the Save the Children, with random assignment across the 58 schools in their programme within Chikowi Traditional Authority.

Power analysis was adjusted for clustering (Hayes and Moulton 2009) and focused on one main outcome of interest: the percentage absenteeism assessed by class attendance registers. The sample size was calculated based on a rate of absenteeism over 12 months.

For attendance, the calculations were performed assuming an average baseline rate of 30% absenteeism and an intraclass correlation of 0.2, 60 children sampled per school (20 children from class 2, 20 from class 4 and 20 from class 6), a sample size of 29 schools with a total of 1740 children in each arm was estimated to provide 80% power to detect a 16% reduction (53% relative reduction) in absenteeism in the intervention group compared to the control group at 5% level of significance. This equates to a total of 58 schools and 3480 children.

However, with allowances made for the possibility of a school not participating in the trial in each arm, a total of 56 schools (3360 children) would allow us to detect 17% reduction in absenteeism in the intervention group compared to the control group with 80% power at 5% level of significance.

vi) Randomization and treatment assignment and allocation

Schools will be randomly assigned to one of two groups. Randomisation will be performed in the presence of the District health and education officers. Each school shall be in a sealed envelope and the envelopes will be picked at random and placed into one of two boxes, A & B (the assignment of group to A and B will have been previously conducted by an individual not involved in the project). The groups shall then be revealed. The public nature of this randomization will ensure transparency of the intervention assignment.

vii) Study intervention

In this study, a school-based malaria control intervention will be provided. The intervention strategy will involve placing PTKs including RDTs for diagnosis of uncomplicated malaria and ACT for treatment in the schools and training two teachers from each school in the use of the items in the PTKs.

The PTK will include: Bandages, gauze, and antiseptic for minor injuries; tetracycline to treat eye infections; paracetamol and aspirin for general aches and pains, oral rehydration salts for diarrhoea, thermometer, gloves and cotton wool, RDTs, lancets, methylated spirit, weighing scales, ACTs for various weight categories, sharps box for lancet disposal, biowaste disposal box, and biscuits (See Appendix 2 for comprehensive list). The PTK box will be a locked wooden box with a thermal blanket for temperature control of the RDTs. Sharp disposals will remain in the box until collection by the health workers. Biowaste will be disposed of in line with the Health technical support services/diagnostics guidelines. In schools that have a separate Head teacher's office, the boxes will be kept securely in the office, however, for single roomed schools, the boxes will be kept at the Head teacher' s house. Only the teachers trained in the PTKs will have access to the box.

All children in the intervention schools are to report to the teachers trained in the PTK use, when in need of medical assistance. In the intervention schools the teacher will follow an algorithm to assist in the decision of what treatment the child may need and whether to perform an RDT for diagnosis of malaria, if suspected. The teacher will be able to then either treat with ACT or refer to the nearest health facility, based on the results of the test.

Two teachers, selected by the head-teacher, from all 29 intervention schools will be trained in the use of the PTKs over four days. The training will be supervised and run by the Health technical support services-diagnostics department of the Ministry of Health. Here they will be trained in the safe and accurate use of an RDT, safe waste disposal, and the treatment

administration of ACTs and additional drugs. A manual previously developed for the PTK implementation, adapted to include the malaria diagnostic and treatment procedures will be provided to all teachers attending the training. Training will follow a structured workshop session developed for Community Health Workers by WHO researchers in Zambia in 2006 (Harvey, Jennings et al. 2008), with the help of WHO RDT "Job Aids" (Appendix 3) . First, a trainer will demonstrate step-by-step how to carry out the test, from opening the test packet to reading the results. Next the trainer will present a module focused on appropriate finger-pricking technique. Participants will then practice the test on one another and receive coaching from the trainer and several experienced assistants. Finally, the trainer quizzes each teacher using photographs to ensure all participants can distinguish between strong positive, faint positive, negative, and invalid results. Participants will be asked to return 2 weeks after training for assessment.

Assessment of teacher RDT use will take place immediately after the training and 2 weeks after training and will focus on two primary outcomes:

a) The ability to carry out the procedure *safely*. That is, without errors which would put individuals at risk of harm from their actions.

b) The ability to carry out the procedure *accurately*. That is, without errors which may lead to either false negatives or false positive results.

The first outcome includes use of clean gloves and a sterile lancet as well as proper disposal of sharps and bio-hazardous materials. The second outcome includes steps essential for test performance: checking the test expiry date, collecting and transferring blood, adding buffer and waiting a sufficient time (15 minutes) before reading test results. In addition to these outcome steps relating to preparation and documentation will be assessed. This includes steps related to prepare the patient, assembling materials and recording results. The checklist used for assessment can be found in Appendix 4.

Training will also comprise instruction on administering treatment, correct dosing, recording all actions taken for each child who reports to the teacher, and will highlight signs and symptoms indicating the need for referral to the local health facility. If the RDT shows a positive result, indicating malaria parasitaemia, the teacher will provide the child with a few biscuits (with milk if possible), and the first dose of the six dose regime of LA. The teacher will then investigate whether the parent lives close to the school and can come in to collect the second dose (to be taken in the evening). If not, the child will be given the dose in the blister pack, cut out from the original packaging, along with a note explaining that the child should take the dose after eating dinner (as the LA can work more effectively if taken with food) and before sleeping. If the child is in class 3 or lower, the LA dose and note will be given to an older sibling in the school, with instructions. In the event of the child not having an older sibling, a relative or neighbour should be called in to hear the instructions as well so that they can deliver the instructions to the parent. Subsequently, morning doses on day two and three will be given at school by the teacher with biscuits and the evening doses, along with instructions to the parents, will be taken home by the child. The following day, the child should bring back the empty blister packaging they took the drugs home in to show the teacher. The teacher will record whether they have seen the empty packaging or not. Each time the teacher delivers the dose over the three days he/she will enquire how the child is feeling and record any reported events such as fever, headache, nausea on an adverse events

monitoring form, in order to monitor whether the child feels worse after taking the LA. Although LA is a very well tolerated drug and no serious side effects are anticipated.

During the training workshop, teachers will receive specific guidelines to follow if the child is not present at school for their second or third day of treatment:

1. The teacher should enquire from the rest of the class as to how far away the child lives from the school and the reason for their absence.
2. The teacher should enquire if the child has a sibling in the same or a different class (classes 4-8). However, if no sibling is identified, a neighbour (in class 4-8) will be selected
3. If the household is close and the child is not absent because they are ill, the sibling or neighbour should be sent to request the child to come in to school for their next dose.
4. If the child's house is not close and/or they are absent because they do not feel well, the sibling/neighbour should be given the morning and evening dose to take home to the child when they go home at lunchtime with a note encouraging the parent to send the child to school the next day if feeling better, and to take them to the health centre if feeling worse. The note will include a few sentences to the health facility nurse to indicate that the child was found positive for P.falciparum infection on (date) and that they have taken X doses of LA and are being referred for further consultation in case the parent decides to take the child to the heath facility. The local health facilities will all be aware of the PTK in schools intervention as they will be advising/ collaborating.
5. However, where possible the teacher should try and follow up individually with the family of the child to check on them if they live close by and are reported as absent due to illness.
6. The teacher should record the actions taken in the pupil treatment records and if the child was ill, the date when their symptoms clear.

Spot-check visits to schools by the research team will monitor whether teachers are following the above procedures.

Data on teacher age, gender, level of formal education, number of years teaching, current school, prior experience treating malaria and prior experience using RDTs will also be collected by means of a self-administered questionnaire (Appendix 5).

The aim of the intervention is to improve prompt access to diagnosis and treatment for malaria amongst school children.

viii) Survey investigations

Seeking consent: All 58 schools will be visited one month prior to initiation of the intervention for the purpose of the intervention to be explained to the head teacher, SMC, PTA and parents. Consent for the intervention in the school will be given at the school level and by the head teacher, PTA and SMC. If parents do not object, their child will be eligible to be assessed using an RDT if they report feeling unwell in school, and results dependant, treated with ACT, by the teacher.

Halliday KE, *et al. BMJ Global Health* 2020; 5:e001666. doi: 10.1136/bmjgh-2019-001666

Study children assessed as part of the evaluation will be randomly selected for follow up from classes 2, 4 and 6. From each class, 30 children will be randomly selected in May to be included in the follow up (evaluation) cross sectional survey in June. Meetings will be held at each school and the parents/guardians of the selected children, plus some reserves shall be invited for a meeting. Parents/guardians of these children will be given information on the outcomes to be measured and the survey procedures. Parents/teachers will be invited to provide individual informed consent for their child's participation. Parents of included children will be asked a series of questions including known parental education, soeciodemographic factors, and location of household (Appendix 6). This information will used to control for potentially confounding variables in the data analysis.

***Evaluation survey procedures*:**

Data on various outcomes will be collected during the school year and at the follow-up surveys in June 2013.

School attendance: Data on attendance over the year will be obtained from class attendance registers kept by teachers. Photographs of the registers will be taken every month to collect the attendance data for entry in the office. Spot checks of attendance will be carried out for quality control.

Daily wellbeing (morbidity): A chart will be put on the wall of classes 2, 4 and 6 in all 58 schools every month. The name of every child in the class will be recorded on the top. The children will draw a smiley, neutral or sad face in the box under their name for each day of the week depending on if they feel very well, average or ill on the day. If the child is absent, a cross is put in the box for that day for that child. These charts will be collected and replaced every month. This will also act to corroborate the school registers in terms of attendance.

School performance: To assess the difference in school performance between the intervention and the control group, tests of attention, literacy and numeracy will be administered at the follow-up survey. These assessments will be similar to those administered in the needs assessment/baseline carried out in 2011.

Health surveys: All study children will be asked to provide a finger-prick blood sample at follow-up to assess malaria parasitaemia, via a blood slide read by expert microscopists, and haemoglobin concentration, via a Hemocue photometer. In addition a series of questions will be asked of each study child and their parent/guardian including: age, fever on the day of the survey, basic household assets indicators, source of potable water in the homestead, use of mosquito nets treated with insecticide (Appendix 7). This information will used to control for potentially confounding variables in the data analysis.

Treatment seeking outside of school: The parents of the study children will be requested to bring their children's health records to the schools for fieldworkers to record the number of visits the child has made to the health facility and the treatments prescribed. Additionally, treatment seeking behavior will be recorded.

Process evaluation: This will aim to (i) investigate community acceptability of teachers trained in using PTKs in schools which include RDTs and ACTs for diagnosis and treatment of uncomplicated malaria; and (ii) document actors external to the intervention which might

impact upon both its implementation and its effectiveness. Such an evaluation will help the interpretation of results and help inform future large-scale implementation of the intervention.

First, a modified stakeholder analysis approach will be adopted to identify and assess the importance of key people, or groups of people who are likely to affect the implementation and longer-term sustainability of the programme (Steckler & Linnan, 2004; (Varvasovszky and Brugha 2000). Key stakeholders are likely to include: teachers, parents, children, community leaders, local health workers and education officers as well as individuals at district and national levels in the Ministries of Education and Health. Stakeholders from both intervention and control schools will be included in order to obtain views on the intervention. An assessment will be made of their importance to the success and sustainability of the programme and data on their views about the programme (e.g. experiences of the intervention, acceptability of the approach, value to individuals & communities, impact on workload) will be collected through a series of focus group discussions as well as semi-structured interviews with people identified as key informants (Bernard, 2006). Appendix 8 is a sample focus group discussion guide for teachers. Each guide will be adapted for each age group and stakeholder.

Second, an analysis will be undertaken of the structural, organizational and management factors that enhance or constrain effective implementation of this expansion on the original PTKs which were initially scaled up. Interviews will be conducted with purposefully selected key stakeholders in order to assess the organization and managerial capacities of the government at national and district levels (Jones, Abeku et al. 2008). These data will be analyzed and interpreted iteratively based on implementation and organizational management theories (Damschroder, Aron et al. 2009) and the developed stakeholder analysis framework.

Feasibility evaluation: The feasibility of the intervention will be measured via a series of random supervisory spot checks made by a health worker to assess teachers' ability to correctly use of the logarithms, RDTs, and provide treatment to children in a professional manner. Monitoring forms will also be checked to ensure that the correct treatment was provided for specific health problems identified and are recorded appropriately (Appendix 9). Teachers will also be given the opportunity to note issues they come across and make suggestions for improvement. A suggestion box will also be placed at the school to allow children to make comments and suggestions relating to the PTKs and school generally.

Cost benefit and cost effectiveness analysis: The evaluation will examine the costs, cost-benefits and cost-effectiveness of placing enhanced PTKs including RDTs and ACTs for diagnosis and treatment of uncomplicated malaria in schools and providing a training programme for teachers in using the PTKs in improving attendance, health and educational outcomes.

The costs of the new PTKs will be assessed from both a provider's and societal perspective. Both financial and opportunity costs will be evaluated using standardized methodologies (Drummond et al., 2005). In addition to estimating the rate of return to school attendance, the cost-benefit analysis will incorporate improved educational performance and daily morbidity into the benefit streams (Jimenez & Patrinos, 2008). Cost per percentage increase in school attendance will be assessed.

ix) Advisory Committee

A local advisory committee has been selected with a view in providing expertise in the principal areas of research that the study will undertake. The selection criterion for members was done with an attempt to ensure a diverse representation of expertise and perspective to help the study achieve its objectives. The advisory committee will consist of several members of the study team as well as additional advisors:

- Mr. John Sande, National Malaria Control Program (Chair)
- Mr. Charles Mazinga, Deputy Director, School Health and Nutrition
- Mr. Gome Jenda, President's Malaria Initiative
- Dr. Don Mathanga, Malaria Alert Centre
- Mr. Ndau, District Malaria Officer, Zomba
- Mr. Phondiwa, District Education Officer, Zomba
- Dr. Chilima, Director of Public Health Laboratory
- Dr. Mwenda, Deputy Director of Health Technical Support Services-Diagnostics
- Mr. Joby George, Save the Children, USA

The Research Advisory Committee's role will be to advise and assist the implementation and progress of the study. It provides initial, regular, and closing advice on all issues related to the study investigations.

x) Quality control

All members of the study team will be trained in the study objectives, methods of effective communication with study participants, and collection of high quality data. Study members will receive additional training specific to the tasks they will perform within the study including interviewing techniques, and clinical and laboratory measurements. A random sample of blood samples will be re-read by experienced technicians and any disparities corrected.

xi) Laboratory procedures

During the follow-up evaluation survey, each child will be asked to provide a finger-prick blood sample for the preparation of a thick and thin blood smear for malaria investigation. Blood smears will be stained with 2% Giemsa for 30 minutes. Parasite densities determined from thick blood smears by counting the number of asexual parasites per 200 white blood cells (or per 500 if the count was less than 10 parasites/200 white cells), assuming a white blood cell count of 8,000/ml. A smear will be considered negative after reviewing 100 high-powered fields. Gametocytaemia will also be determined from thick smears. Thin blood smears will be reviewed for species identification. Two independent microscopists will read the slides, with a third microscopist resolving discrepant results.

The same finger prick sample will be used at the point of survey to record hemoglobin concentrations using a portable HemoCue photometer (HemoCue, Angelhom, Sweden). Axillary temperature will be measured with a digital thermometer. Children with fever (≥ 37.5°C axillary) or history of fever in the previous 24 hours will be tested with a malaria RDT (Bioline Malaria Ag Pf/Pan®), and treated according to national guidelines. Height will be measured to the nearest 0.1cm using a Leicester portable fixed based stadiometer and weight will be measured to the nearest 0.1kg using an electronic balance.

Halliday KE, *et al. BMJ Global Health* 2020; 5:e001666. doi: 10.1136/bmjgh-2019-001666

VI. DATA MANAGEMENT

Data entry and storage

All questionnaires will be transferred to Zomba for review, data entry, and storage. Data will be entered by two independent clerks, will be verified for data entry errors, and corrected from the original questionnaire using customized software (Microsoft Visual FoxPro® 6.0) and verified for accuracy. Study records will be stored securely in the Save the Children Zomba office.

Data analysis

Data on community and stakeholder views about the feasibility and impact of the program (e.g., experience of the intervention, acceptability of the approach, value to individuals & communities, impact on workload) will be collected through a series of focus group discussions as well as semi-structured interviews with people identified as key informants. Discussions and interviews will be transcribed and translated, and content analysis using Nvivo 8 (QSR International, Melbourne, Australia) will be undertaken to identify themes based on people's experience and involvement in the intervention.

As this is an effectiveness study all analyses will be on the basis of intention-to-treat. To assess the success of randomization baseline characteristics between the two randomized groups will be compared. To assess the impact of the intervention, methods appropriate for cluster-randomized trials will be used (Hayes and Moulton 2009). The prevalence, or mean, in each school will be calculated and the unadjusted risk ratio (RR), or mean difference (intervention-control), estimated in each stratum. An overall estimate of the effect of the intervention will be obtained by taking a weighted average of the stratum-specific estimates, the weights proportional to the number of schools per stratum, and 95% confidence intervals will be adjusted for observed between-school variance. Formal hypothesis testing will be undertaken using stratified unpaired t-tests. The impact of the intervention will be estimated for education outcomes and health outcomes in separate multiple regression models. Statistical analysis will be carried out using STATA® software, version 12.0. (Stata Corporation, Texas, USA).

VII. ETHICAL CONSIDERATIONS

Ethical approval

The study will seek approval from the National Health Sciences Research Committee of Malawi and the London School of Hygiene and Tropical Medicine.

Informed consent process

Prior to the onset of the school intervention, all schools will be visited and meetings will be held with the head teacher, school management committee (SMC), and parent teacher association (PTA) to describe the purpose of the school intervention, the procedures to be followed, and the risks and benefits of participation. Consent for the intervention in the school will be given at the school level and by the head teacher, PTA, and SMC. Parents/guardians of children in classes 1-8 will be informed at the meeting that if they would like to opt out of the study they can sign an "opt out" form (Appendix 10 & 11). If they do not object, their child will be eligible to be assessed using an RDT, and results dependant, treated, by the teacher if they report feeling unwell in school. In the schools randomly selected to receive the intervention, consent will be sought from each of the two teachers selected to be trained as

Halliday KE, *et al. BMJ Global Health* 2020; 5:e001666. doi: 10.1136/bmjgh-2019-001666

PTK dispensers. Prior to consenting, prospective teachers will be given an information sheet (Appendix 12 & 13) that includes an introduction, the purpose of the study, and what their participation will entail. Teachers will have the chance to ask questions and will be informed that their participation in the study is completely voluntary and that they may withdraw from the study at any time.

For the follow up survey, children will be randomly selected from classes 2, 4 and 6. From each class, 30 children will be randomly selected in May to be included in the follow up cross sectional survey in June. Meetings will be held at each school and the parents/guardians of the selected children, plus some reserves shall be invited for a meeting. Parents/guardians of these children will be given information on the outcomes to be measured and the survey procedures. The parents or guardians will be asked to sign consent for their child (if aged under 18 years) to participate in the research study (Appendix 14 & 15). For those parents who do not attend these meetings, follow-up will be made through community leaders and household visits. If a parent, guardian or older child is unable to read or write, his/her fingerprint will be used in substitute for a signature, and a signature from a witness to the informed consent discussion will be obtained. Parents and guardians will have the chance to ask questions and will be informed that participation of their child(ren) in the study is completely voluntary and that they may withdraw from the study at any time.

Written consent to participate in the stakeholder interviews and focus groups discussions will be sought (Appendix 16 & 17).

Training for those involved in administering consent
All fieldworkers will undergo training prior to the study. Training will educate fieldworkers on the purpose of the study, the importance of consent and how to administer both the consent forms and questionnaires. While in the field, fieldworkers will have continuous contact through the use of a mobile phone with the team leaders in case of any queries.

Benefits
This study has been designed to address several areas of major public health and educational significance for Malawian schoolchildren. If a relationship demonstrates that malaria treatment in schools improves school attendance and other health and educational outcomes, is feasible and cost effective, the NMCP and national SHN program are keen to scale this intervention up to all schools in Malawi as they did previously with the PTKs in 2007. The results of this study will also help inform other national SHN and malaria programs seeking evidence to guide their programming.

Children randomized to the PTK intervention arm will benefit from free medical treatment provided by the trained teachers. RDTs will be performed for school children that have a fever; those children found to be positive with malaria parasites can immediately receive treatment, or be referred to the nearest health facility. Children found to be negative for malaria parasites will be treated with paracetamol.

Risks
Parent/guardians and children will be informed of all potential risks. The collection of finger-prick blood samples may lead to transient bleeding, minor temporary discomfort or pain for children. Precautions will be taken to avoid bleeding by immediate application of sterilized cotton wool and pressure at the prick site. Risks of infection will be minimized by using

disposable sterile lancets, one for each child to avoid cross contamination/transmission of infectious agents.

The study involves specific risks from the artemisinin-based combination therapy (ACT) drugs. ACTs are remarkably well tolerated in humans, with no serious adverse events or significant toxicity reported.

Community engagement
Prior to the onset of the study, meetings will be held with officials from the District Education Office and the District Health Office to sensitize them about the study and plans for training and assessment. Similar meetings will also be held with officials at the national level in Lilongwe.

Upon completion of the study, a two page 'Research Brief' will be developed. In addition, a one-day feedback meeting with headteachers of participating schools and the district health and education staff will be held, where the results of the study will be presented and discussed. Headteachers will be asked to distribute the Research Brief to interested parents.

Confidentiality
Participants, parents and guardians will be informed that participation in a research study may involve a loss of privacy. All records will be kept confidential. Participants will be identified primarily by their study number and patient names will not be entered into the computerized database. No individual identities will be used in any reports or publications resulting from the study.

VIII. DISSEMINATION OF RESULTS

The results will be disseminated primarily to the National Malaria Control Program (Ministry of Health), and the SHN Program (Ministry of Education, Science and Technology), as well as to the District Health Office and District Education Office, and other interested stakeholders at national and local level. This study forms part of the evidence-base required by the government of Malawi to inform the decision as to the appropriate malaria control strategy in schools. It is also anticipated that the results will be presented at appropriate national and international scientific meetings and several papers will be written and submitted to peer-reviewed scientific journals for publication. This study will contribute to the global evidence base on malaria control in schools which is still weak and inform malaria control in schools strategies in other countries in the region.

Halliday KE, *et al. BMJ Global Health* 2020; 5:e001666. doi: 10.1136/bmjgh-2019-001666

IX. TIMEFRAME

**2012**	Jun	Jul	Aug	Sep	Oct	Nov	Dec
Assess acceptability of community stakeholders + teachers of teacher-led RDTs	■	■					
Develop and test tools for training materials for teachers and monitoring tools		■	■				
Conduct a 3 day training workshop for 2 teachers from 29 intervention schools on the PTK incl. RDT use and ACT treatment				■			
Assess teachers retention of diagnosis, RDT use and treatment 2 weeks following training				■			
Survey of children's KAP of malaria, school attendance, family's bednet use.				■	■		
Teachers implement the PTK and malaria prevention education in all schools and the diagnosis+treatment of fever cases in 29 schools					■	■	■
**2013**	Jan	Feb	Mar	Apr	May	Jun	Jul
Teachers implement the PTK and malaria prevention education in all schools and the diagnosis+treatment of fever cases in 29 schools	■	■	■	■	■		
Follow-up health surveys, attendance surveys, collect health record cards.						■	
Analysis, write up and dissemination							■

X. BUDGET

Item	No	Days	Rate	Amount (US$)
a) Principal investigators	2	7	200	2800
b) Fieldworker salaries	4	100	15	6000
c)Lab technicians and nurses	10	6	15	900
d)Education assessors	8	14	12	1350
e)Introductory school visits and meetings	58	1	15	870
f) Residential Training costs	60	4	23	5520
g) Supplies and survey materials (PTKs)				10000
h) Local travel (+drivers)				2500
i) Data entry				1000
j)Contigencies				800
k) Ethical committee 10% of budget				3174
Total				34914

21

XI. BUDGET JUSTIFICATION

Principal investigators. This includes perdiems and travel of PI visits to the field.

Fieldworker salaries. This includes the costs of the fieldworkers who will be monitoring the intervention throughout the study period, eg collecting and replacing monthly morbidity charts, carrying out attendance spot checks and health facility staff who will aid in providing support to the schools throughout the intervention.

Lab technicians and nurses. This is estimated cost of employment of district technicians and nurses for the follow up health surveys.

Education assessors. This is the estimated cost of employment of fieldworkers for the education surveys and the questionnaires at the follow up surveys.

Introductory school visits and meetings. During the study preparation an investigator will need to meet with local officials and school head teachers. School sensitisation meetings will be held at each school with parents. The fieldworkers will be conducting these meetings.

Residential training and costs. The study involves training two teachers per school on the use of PTKs by members of the Health technical support services/diagnostics. This requires an initial teacher training over a four-day period followed by ongoing monitoring and support. All costs for travel and accommodation have been estimated using standard Save the Children mileage and per diem charges.

Supplies and survey materials This includes the costs of the PTK supplies that will be given to each of the 29 intervention schools. In addition all of the materials for the monitoring and evaluation of the PTK intervention required by Save the Children office in Zomba are included here, such as any survey instruments that require purchasing, photocopying, stationary and communication. The estimates are based on routine costs charged at the unit.

Local travel (+drivers) This includes the costs for the transport of the supervisors and fieldworkers to visit the schools during the intervention period to monitor progress as well as the transportation of the survey teams during the follow up evaluation surveys.

Data entry The estimated cost of the creation of the databases and the double entry of the routine monitoring data collected as well as the evaluation data collected in the follow up surveys.

Contingencies This includes other costs that may need to be added during the study period.

Halliday KE, *et al. BMJ Global Health* 2020; 5:e001666. doi: 10.1136/bmjgh-2019-001666
