# Supplementary material for: Impact of school-based malaria case management on school attendance, health and education outcomes: a cluster randomised trial in southern Malawi
Source: BMJ Glob Health. 2020 Jan 14;5(1):e001666. doi: 10.1136/bmjgh-2019-001666 (PMC7042571; doi:10.1136/bmjgh-2019-001666)
Supplement: Supplementary data [file bmjgh-2019-001666supp003.pdf]

Impact of a primary school-based malaria case management programme on school attendance and health in southern Malawi: a cluster randomised trial - Supplementary information

Supplementary Figure S1: (a) The location of TA Chikowi, Zomba in southern Malawi and (b) the location of the 58 study schools, in both intervention and control groups, and linked health facilities.

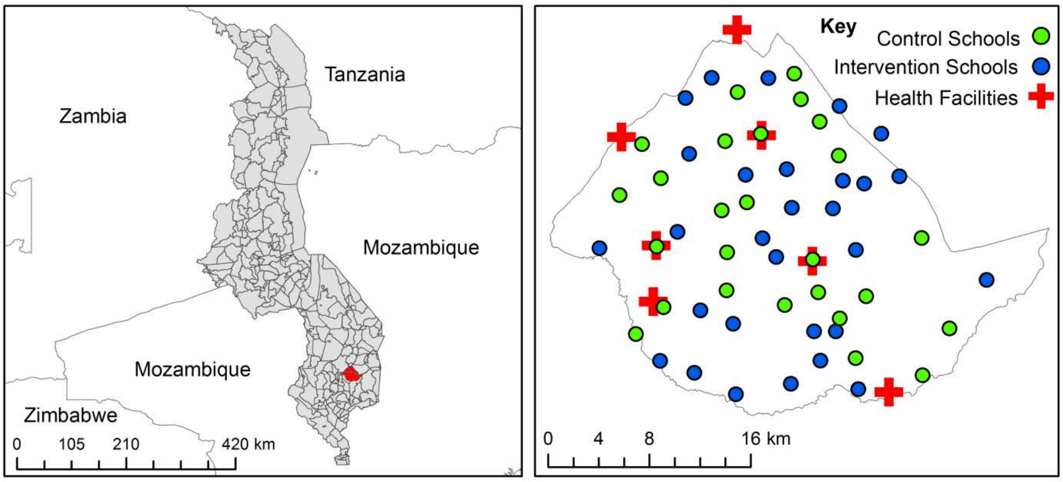

Supplementary Table S1: percentage absenteeism at each spotcheck by study group. Results are presented adjusted for clustering at the school level.

Spotcheck	N control	Absent	% absent in control	N intervention	Absent	% absent in intervention	Odds ratio (95% CI)
1 May/Jun 14	2,492	799	32.06 (26.64 – 37.49)	2,692	823	30.57 (25.23 – 35.92)	0.96 (0.75 to 1.24)
2 July 14	1,135	221	19.47 (15.71 - 23.23)	887	193	21.76 (17.31 - 26.21)	1.10 (0.79 to 1.55)
3 Sept 14	2,395	396	16.53 (12.92 – 20.15)	2,292	456	19.90 (16.23 – 23.56)	1.31 (1.00 to 1.72)
4 Nov 14	2,363	385	16.29 (12.48 – 20.11)	2,351	454	19.31 (15.50 – 23.12)	1.27 (0.97 to 1.66)
5 Jan/Feb 15	1,142	315	27.58 (22.38 – 32.79)	1,231	343	27.86 (22.92 – 32.81)	0.96 (0.71 to 1.31)

Supplementary Table S2: ICCs presented for the primary and secondary outcomes

Outcome	Number of clusters	Unadjusted model			Adjusted model		
		Average cluster size	Range	ICC	Average cluster size	Range	ICC
Daily attendance	58	155.5	97 – 171	0.027	149.5	88 – 169	0.027
Parent reported attendance (days absent)	58	8.1	2 – 24	0.127	7.9	2 – 23	0.110
Spotchecks	58	100.0	62 – 110	0.036	94.9	56 – 107	0.036
Anaemia	58	60.5	37 – 77	0.039	59.3	36 – 77	0.039
Infection with *Plasmodium*	58	60.4	37 – 77	0.146	59.2	36 – 77	0.090
Child-reported well-being	57	69.6	0 – 102	N/A*	67.4	0 – 101	N/A*
Standardised literacy score	58	47.3	1 – 71	0.124	46.3	1 – 71	0.108
Standardised numeracy score	58	48.0	1 – 73	0.143	47.0	1 – 73	0.116
Parasite density (log count)	57	9.6	1 – 27	0.024	9.4	1 – 27	0.013
Haemoglobin (mg/l)	58	60.6	37 – 77	0.044	59.3	36 – 77	0.048

*ICCs are not available for ordinal logistic regression models
